# Concentration optimization of combinatorial drugs using Markov chain-based models

**DOI:** 10.1186/s12859-021-04364-5

**Published:** 2021-09-21

**Authors:** Shuang Ma, Dan Dang, Wenxue Wang, Yuechao Wang, Lianqing Liu

**Affiliations:** 1 State Key Laboratory of Robotics, Shenyang Institute of Automation, Chinese Academy of Sciences, Shenyang, China; 2 Institutes for Robotics and Intelligent Manufacturing, Chinese Academy of Sciences, Shenyang, China; 3 University of Chinese Academy of Sciences, Beijing, China; 4 Faculty of Medical Devices, Shenyang Pharmaceutical University, Shenyang, China

**Keywords:** Combinatorial drug optimization, Markov chain, Transition probability, Stationary balance distribution, Combinatorial therapy

## Abstract

**Background:**

Combinatorial drug therapy for complex diseases, such as HSV infection and cancers, has a more significant efficacy than single-drug treatment. However, one key challenge is how to effectively and efficiently determine the optimal concentrations of combinatorial drugs because the number of drug combinations increases exponentially with the types of drugs.

**Results:**

In this study, a searching method based on Markov chain is presented to optimize the combinatorial drug concentrations. In this method, the searching process of the optimal drug concentrations is converted into a Markov chain process with state variables representing all possible combinations of discretized drug concentrations. The transition probability matrix is updated by comparing the drug responses of the adjacent states in the network of the Markov chain and the drug concentration optimization is turned to seek the state with maximum value in the stationary distribution vector. Its performance is compared with five stochastic optimization algorithms as benchmark methods by simulation and biological experiments. Both simulation results and experimental data demonstrate that the Markov chain-based approach is more reliable and efficient in seeking global optimum than the benchmark algorithms. Furthermore, the Markov chain-based approach allows parallel implementation of all drug testing experiments, and largely reduces the times in the biological experiments.

**Conclusion:**

This article provides a versatile method for combinatorial drug screening, which is of great significance for clinical drug combination therapy.

**Supplementary Information:**

The online version contains supplementary material available at 10.1186/s12859-021-04364-5.

## Background

In the practice of clinical treatment, a single drug often fails to achieve the desired efficacy because the single drug in general aims at a single target of diseased cells and cannot remedy all aberrantly functioning pathways because of the robustness of organisms. The drug may also have poor safety profiles owing to various factors [1], including compensatory changes in cellular networks upon drug stimulation [2], redundancy [3], crosstalk [4], and off-target activities [5]. In contrast, drug mixtures are generally more effective than single effectors because multiple drugs simultaneously act on different pathways and cell targets, potentially leading to higher efficacy and lower toxicity because of drug synergy [6]. Therefore, in the clinical treatment of complex diseases, such as parasitic nematode infections or herpes simplex virus (HSV), a variety of drugs have been used in combination for treatment improvement [7]. The infection of parasitic nematodes (or roundworms) poses a serious safety hazard to humans and livestock [8], and the anthelmintics (or antinematode drugs) are highly susceptible to drug resistance. It has been proved that a variety of combinations of multiple anthelmintic drugs, rather than a single medicine, can enhance the deworming effect [9]. In the case of the eradication of wild-type *Caenorhabditis elegans* worms, it is more effective to use four combinatorial drugs (levamisole, pyrantel, tribendimidine, and methyridine) than single drugs [10]. Traditional treatments of HSV-I, one of the most common sexually transmitted infections, often include virus-specific drugs, which are effective at the beginning but exhibit limited long-term efficacy as drug-resistant strains develop. However, a combination of six drugs (IFN-α, acyclovir, IFN-γ, ribavirin, IFN-β and TNF-α) was demonstrated to be the most promising therapy for the reason that the drugs in the combinatorial treatment can act simultaneously on the multiple pathways and cellular protein complexes, and, therefore, regulate all relevant pathways, potentially blocking HSV-I replication [11]. Combined use of multiple drugs is also a common practice in the treatment of cancers to achieve higher efficacy and potency. For example, in the treatment of non-Hodgkin’s lymphoma, the drugs, pirarubicin, velet, cytarabine and prednisone, are usually used in combination, which the chemotherapy effect is remarkablely enhanced [12].

However, owing to the inherent complexity of biological systems and internal structure of cells and, particularly, to the huge searching space, it is extremely challenging to effectively and efficiently to determine the optimal drug mixture from all possible drug combinations through trial and error. For example, there are *n* drugs and each drug has *m* concentration candidates, it is necessary to find the optimal drug mixture in the space of $m^{n}$ combinations. Obviously, as the types of drug increase, the number of combinations increases exponentially, and it is impossible to test all cases of drug combinations because it takes a considerable amount of time to perform the testing experiments. Therefore, it is important to explore how to reduce the number of experiments and predict the optimal combinatorial drug concentration accurately and quickly.

For these reasons, the optimization of drug combination has attracted considerable attention in recent years, and several methods for predicting the optimal combinatorial drug concentrations have been proposed [13–20]. A feedback system control (FSC) method was developed to search for optimal synergistic combinatorial drugs for the treatment of diseases [16]. The FSC method starts with a set of initial concentrations of combinatorial drugs with defined drug doses, and the efficacies of the combinatorial drugs on the cells at the given concentrations are evaluated according to the phenotypic output response of the cells. Then, the next predictions of the concentrations of drug mixtures are conducted based on the previous drug testing results with a certain searching algorithm, such as the Gur game (GG) algorithm, modified Gur game (MGG) algorithm, differential evolution (DE) algorithm, streamlined-feedback system control (s-FSC) algorithm, continuous adaptive population reduction (CAPR) method, and the FSC method iteratively approaches a globally optimal combinatorial drug mixture [19]. However, in some cases, these algorithms may degrade the overall performance of FSC owing to the inherent shortcomings of these algorithmic frameworks [17–20]. FSC with the GG and MGG algorithms often falls in oscillatory curves instead of giving a convergent output. It converges too early to a local extremum with the DE algorithm, thereby forming a premature convergence phenomenon, and it lacks a unified parameter controlling strategy with the CAPR algorithm to satisfy various applications. As the search process of s-FSC method is based on an ‘iterative cycle’, searching for the optimal concentration requires more iterations. Furthermore, the FSC iterates its searching process, in which the next iteration requires biological experiments with the predicted combinatorial drug doses for further evaluation and prediction. Thus, the optimization of combinatorial drugs with FSC is quite inefficient because a significant amount of time is spent on the testing experiments.

Markov chain is one of the most important and fundamental algorithms in the field of machine learning, and is being used widely in the analysis of biological data and in bioinformatics area. It is novel to use the Markov chain method to model the combinational drug optimization and search for the optimal concentration combination. In this paper, an optimization method based on Markov chain models is proposed to search for optimal combinatorial drug concentrations with excellent performance. In this method, the searching process of the optimal drug concentration is converted into a Markov chain with $N = m^{n}$ state variables representing all possible drug combinations, where *n* refers to the number of drugs, and *m* is the number of discretized concentrations for each drug. This Markov chain can be depicted by a network of *N* nodes in the space of $R^{n}$, where the nodes refer to the state variables. Assuming that all the possible drug combinations have equal probability to be the optimal mixture without having prior knowledge about the efficacy of the drug mixtures, a matrix of transition probability can be initialized so that the stationary distribution vector of the Markov chain has an equal value of 1/*N* for all its states. Then the searching process for the optimal combinatorial drug concentrations is equivalent to updating the transition probability matrix and seeking the the state with the maximum value in the stationary distribution vector.

The proposed method was validated by both simulation and biological experiments. In the simulation experiments, the proposed Markov chain-based method was compared with the five benchmark algorithms (GG, MGG, DE, CAPR and s-FSC) in the FSC framework. In biological experiments, the survival rate of cells under two combinatorial drugs is regarded as the response function, and the Markov chain-based method was compared with GG and MGG in FSC. The results of the simulation and biological experiments prove that the algorithm based on the Markov chain outperforms the selected benchmark algorithms in terms of accuracy and efficiency. In summary, this study provides a versatile, novel method for efficiently optimizing combinatorial drug concentrations, and the work is of great significance for clinical drug combination therapy.

The remainder of this article is organized as follows. First, the preliminary theories of the Markov chain are discussed briefly, and the Markov chain-based method is presented. Then the simulation and biological experiments are described, and the experimental results are discussed to compare the performance of the proposed Markov chain-based method with other benchmark algorithms. In the last, we conclude this article.

## Methods

In this section, first, some basic theories of the discrete-time Markov chain are briefly reviewed. The optimization problem of combinatorial drug therapy is formulated with assumptions, and the general idea of the Markov chain-based approach to the optimization of drug combinations are described. The detailed algorithms in the cases of one drug and two drugs are given in Additional file 2: Fig. S1, Additional file 3: Fig. S2, Additional file 4: Fig. S3, Additional file 5: Fig. S4, Additional file 6: Fig. S5, Additional file 7: Fig. S6.

### Markov chain theory

A Markov chain is a special kind of Markov stochastic process with a set of discrete states. It starts in one of these states and moves successively from one state to another, satisfying the Markov property. Markov chains are a mathematical model to describe a process in which the next state of the system depends only on the present state, and not on the preceding states. In other words, the process loses its memory of the past over time.

**Definition of a Markov chain: **When $\left\{ {X_{n} ,n \ge 0} \right\}$ is a random sequence taking values in a finite or countable discrete set, where $\Phi = \left\{ {1,2, \ldots , N} \right\}$ or $\Phi = N$ typically, the process $X\left( n \right) = X_{n}$ for $n = 1,2, \ldots$ is a Markov chain if

1 $$P\left( {X_{n + 1} = s_{n + 1} \left| {X_{n} = s_{n} ,X_{n - 1} = s_{n - 1} , \ldots ,X_{0} = s_{0} } \right.} \right) = P\left( {X_{n + 1} = s_{n + 1} \left| {X_{n} = s_{n} } \right.} \right)$$

where $n \ge 0$ and $s_{n + 1} ,s_{n} ,s_{n - 1} , \ldots ,s_{0} \in \Phi$, and the values taken by the random variables $X_{n}$ are called the states of the chain. Moreover, if the transition probability $P\left( {X_{n + 1} = s_{n + 1} \left| {X_{n} = s_{n} } \right.} \right) = p_{ij}$ for $s_{n + 1} = j$ and $s_{n} = i$ is independent of $n$, then $P = \left\{ {p_{ij} } \right\}$ is called the transition probability matrix.

**Stationary distribution:** For a Markov chain with the transition probability matrix $P = \left\{ {p_{ij} } \right\}$, a probability distribution vector $\pi$ is called a “stationary distribution” if $\pi$ has entries $\left\{ {\pi_{j} \ge 0,j \in \Phi } \right\}$ such that the following conditions hold.

$$\left\{ \begin{gathered} \pi = \pi P \hfill \\ \sum\nolimits_{j \in \Phi } {\pi_{j} = 1} \hfill \\ \end{gathered} \right.$$

where $\pi = \pi P$ is called the “balance equation.”

### Assumptions

Before introducing the combinatorial drug optimization method based on the Markov chain, it is assumed that there are two assumptions:

- With the slow change in the concentration of the combination drugs, the effect of the drug on the experimental subject also changes smoothly.
- The number of drug combinations is limited.

The above two assumptions are reasonable for the optimization of combined drug concentrations. The organism does not change dramatically under smooth input from the outside world. In the experiment, the concentration of the drug combination is a few discrete points. Under the above two assumptions, the combinatorial drug optimization problem can be expressed using a finite-state Markov chain.

### Method description

First, a general example is depicted to illustrate the main idea of the method. Suppose that there are n kinds of drugs and each drug has m possible concentrations, then the state space ${\Phi } = \left\{ {1,2, \ldots ,m^{n} } \right\}$ represents the set of *m*^*n*^ combinatorial drug concentrations in an ascending order. Our goal is to find the optimal concentration from the state space. Here, the drug response function or death rate of cells can be represented by a normalized function $f\left( x \right) \in \left[ {0,1} \right]$ for $x \in \Phi$. The higher value of the $f\left( x \right)$, the better effect of the drug combination at the corresponding concentration, leading to higher cell death rate. $f\left( x \right)$ = 0 means that the drug combination at the concentration level $x$ is completely ineffective while $f\left( x \right)$ = 1 indicates that the drug combination achieves its best treatment efficacy. Our aim is to find the best concentration $x^{*}$ for drug combination with the maximum value of the objective function $f\left( x \right)$ as follows:

3 $$x^{*} = argmax\, f\left( x \right),\;x \in \Phi$$

**Fig. 1:**
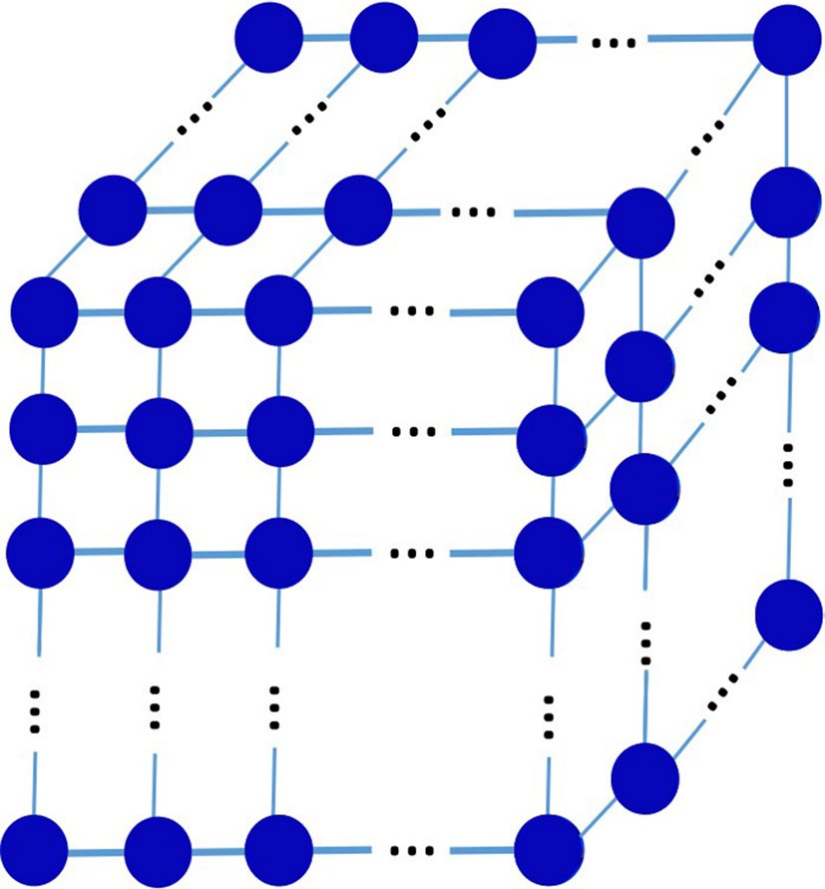
State-transition diagram of a Markov chain with ${m}^{3}$ states

As shown in Fig. 1, in the case of three drugs, it is necessary to construct a three-dimensional network structure of Markov chain. Each drug has *m* concentration levels and a total of $m^{3}$ concentration combinations constitutes the state space $\Phi = \left\{ {1,2, \ldots ,m^{3} } \right\}$. The states in $\Phi$ represent the drug combination of different concentrations. For any $i,j \in \Phi$; if $f\left( i \right)$ > $f\left( j \right)$*,* it is implied that the efficacy of the drug combination at the concentration level $i$ greater than that at concentration level $j$. Likely, in the general case of *n* drugs, an *n*-dimensional network of Markov chain can be constructed, and the state space ${\Phi }$ consists of $m^{n}$ states if each drug has *m* concentration levels.

In order to search for the optimal drug combination, a key assumption with the Markov chain model is that, for any state $x\left( t \right)$, the state $x\left( {t + 1} \right)$ at the next moment always comes from the current state $x\left( t \right)$, and the states have a larger probability shifting to the direction with a larger objective function. In other words, at the step $t$, if the objective function for the state $x\left( t \right)$ is $f\left( {x\left( t \right)} \right)$, then the state $x\left( t \right)$ can select to transfer to its adjacent states to obtain the next state by comparing its function value with those at the adjacent states and choosing the state with a relatively higher objective function value for the next step. The benefits of this approach are obvious. As $t$ approaches infinity, the state transfers to the optimal state x*, which means that the probability at the global maximum of the objective function is the greatest.

Reconsidering the Markov chain model described above, from the state transition diagram shown in Fig. 1, it is obvious that it is a random walk with any two adjacent states. Searching for the optimal drug concentration is equivalent to seeking the state with the largest steady-state probability in the stationary distribution. Therefore, a transition probability matrix *P* of $m^{n} \times m^{n}$ is initialized and then updated iteratively for searching for the optimal drug combination with the Markov chain model. Firstly, according to the initial state, the corresponding transition probability matrix *P* is constructed, and two suitable experimental points are selected from the state space. Secondly, the transition probability matrix is updated and then the balance equation is solved to achieve the stationary distribution. After multiple iterations, the algorithm converges with a predefined criteria, and the maximum value in its stationary distribution is the corresponding optimal state sought, that is, the optimal combination of drug concentration levels.

It is noteworthy that the initialization of the transition probability matrix is not unique. Without having prior knowledge about the efficacy of the drug mixtures, it is reasonable to assume that all the possible drug combinations have equal probability to be the optimal mixture and a matrix of transition probability can be initialized so that the stationary distribution vector of the Markov chain has an equal value of $1/N$ for all its states. In this study, the transition matrix is initialized such that, on the network, every pair of adjacent states has an equal transition probability to move back and forth between each other, and every state has the same transition probability to move to all its adjacent states. In particular, the state on the edge of the network has a certain probability to go back to itself. Then, the Markov chain-based approach to optimizing the combinatorial drugs turns into a process of repeatedly updating the transition matrix by comparing the efficacies of pairs of adjacent drug combinations and then computing the corresponding stationary distribution vector until a certain convergent criterion is satisfied. The steady state that has the maximal distribution probability is referred to as the optimal drug combination.

The general procedure of the optimization algorithm for combinatorial drugs based on the Markov chain model is described as follows:

- *Step 1*: The Markov chain and the corresponding transition probability matrix are initialized according to the numbers of drugs and concentration levels.
- *Step 2*: Suitable adjacent combinations of experimental points are selected.
- *Step 3*: The transition probability matrix of the Markov chain is updated according to the difference in the drug response functions at the corresponding suitable experimental points.
- *Step 4*: The corresponding stationary distribution is solved according to the updated transition probability matrix using the balance equation.
- *Step 5*: It is determined whether the stationary distribution converges. If it converges, the algorithm stops; otherwise, it returns to the second step, or, when the predetermined number of iterations is reached, the algorithm stops.

A single drug and two kinds of drugs are taken as examples to introduce the searching algorithm we proposed in Additional file 2: Figs. S1–S6.

## Simulation experiments and discussion

Simulation experiments were conducted to compare the performances of the Markov chain-based algorithm and five benchmark algorithms: GG algorithm, MGG algorithm, DE algorithm CAPR method and s-FSC method. The principle of these algorithms and the relevant control parameters selection for these algorithms are introduced briefly in Algorithm 1. The simulation experiments were implemented with the response functions in the cases of single drug, two drugs and three drugs respectively.

### Predicting the optimal concentration of single-drug

**Fig. 2:**
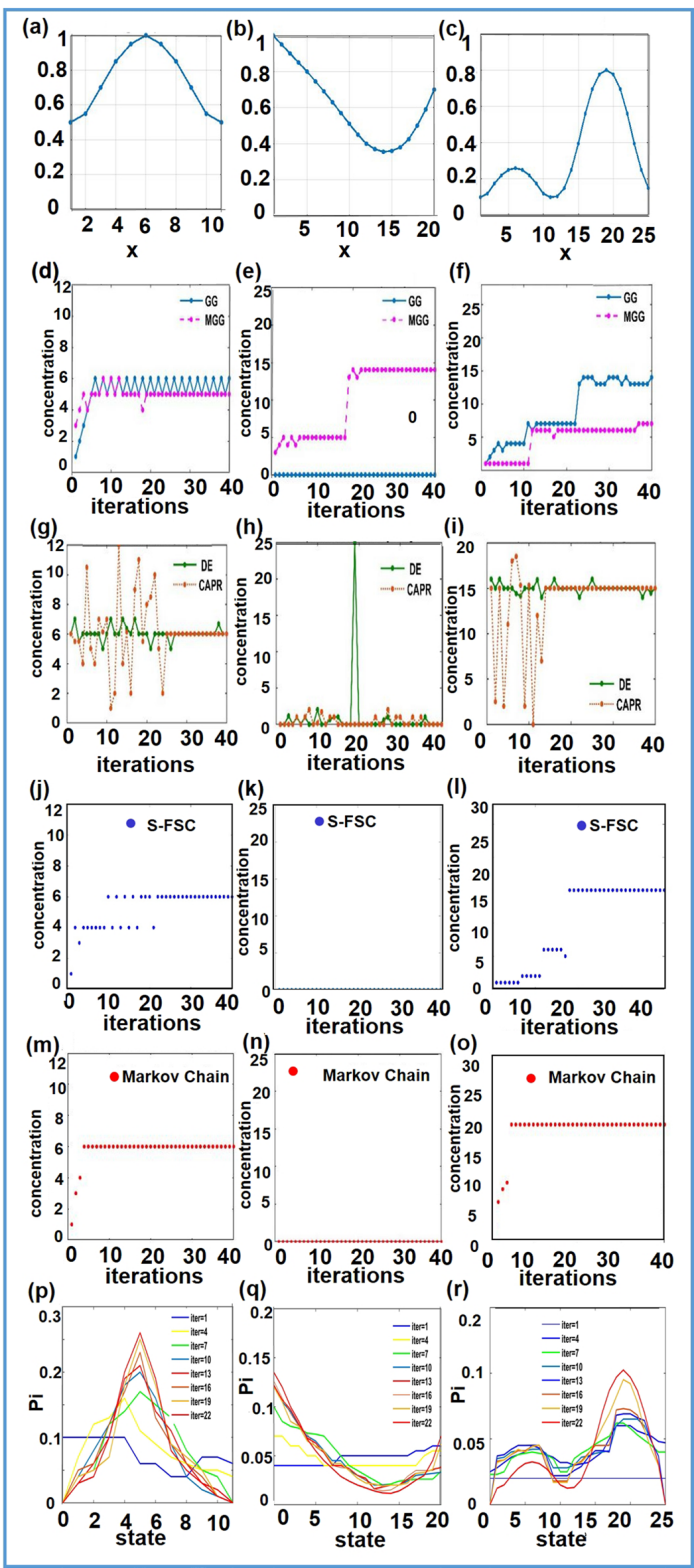
Three drug response functions and numerical simulations using six different algorithms. **a**–**c** Drug response functions; **d**–**f** GG algorithm and MGG algorithm; **g**–**i** DE algorithm and CAPR algorithm; **j**–**l** s-FSC algorithm; **m**–**o** Markov-chain-based algorithm; **p**–**r** the stationary distributions

Three drug response functions of single drug are used to compare the performances of the Markov chain-based algorithm and the benchmark algorithms, including GG algorithm, MGG algorithm, DE algorithm, CAPR algorithm and s-FSC algorithm. And the three response functions of single drug, corresponding to the curves in Fig. 2a–c, are defined as below respectively:

$$\begin{aligned} & f_{1} (x) = - 0.000845x^{4} - 0.02028x^{3} + 0.1415x^{2} - 0.2374x + 0.6136, \, (1 \le x \le 11); \\ & f_{2} (x) = 0.000006498x^{4} + 0.0001245x^{3} - 0.004669x^{2} - 0.2374x + 1.0016, \, (1 \le x \le 20); \\ & f_{3} (x) = - 0.000000006413x^{8} + 0.0000006606x^{7} - 0.00002695x^{6} + \\ & \quad 0.0005516x^{5} - 0.005942x^{4} + 0.0328x^{3} - 0.08933x^{2} + 0.1461x + 0.01262,(1 \le x \le 25); \\ \end{aligned}$$

In the simulations, it is expected to find the global maximum points for the drug functions. As shown in Fig. 2, the GG and MGG algorithms can not successfully find the global optimum points for all three drug response functions, and sometimes oscillate around some states (Fig. 2d) or stays in a suboptimal states (Fig. 2e, f). The DE algorithm and the CAPR algorithm not only fail to find the global maximum for some functions, but also take much more time to achieve the solutions (Fig. 2g–i). The s-FSC algorithm can find the optimal point but sometimes it need more steps (Fig. 2j–l). In the contrast, the proposed Markov chain-based approach always succeeds in obtaining the global optimal state with less iterations (Fig. 2m–o). A further comparison of the Markov chain-based algorithm and the five benchmark algorithms was made using the success rate and the number of iterations, as shown in Table 1, which demonstrate that the Markov chain-based algorithms is more reliable and efficient than the benchmark methods.

**Table 1:** Performance comparison of six algorithms in simulations

	GG		MGG		DE		CAPR		s-FSC		Markov chain	
	Success rate	# of iters	Success rate	# of iters	Success rate	# of iters	Success rate	# of ites	Success rate	# of ites	Success rate	# of iters
*f*_1_												
λ = 0.95	0.67	30.6	0.76	25.2	0.72	34.4	0.89	21.2	0.92	27.5	1.00	8.6
*P* = 5%	0.88	18.2	0.89	20.3	0.69	30.3	0.87	17.5	0.91	22.5	1.00	5.3
*f*_2_												
λ = 0.95	0.68	32.6	0.70	25.2	0.65	28.7	0.91	22.2	0.96	12.6	1.00	9.2
* P* = 5%	0.89	17.2	0.80	20.3	0.72	23.3	0.92	15.6	0.95	10.6	1.00	7.3
*f*_3_												
λ = 0.95	0.60	27.6	0.78	29.2	0.65	27.7	0.91	28.2	0.97	29.2	1.00	17.2
*P* = 5%	0.84	18.2	0.88	21.3	0.75	30.3	0.92	19.6	0.96	22.5	1.00	12.3
*f*_4_												
λ = 0.95	0.82	85.6	0.92	60.2	0.78	88.4	0.85	78.2	0.88	71.2	1.00	43.6
*P* = 5%	0.95	35.2	0.99	25.3	0.62	43.3	0.72	44.5	0.78	40.5	1.00	22.3
*f*_5_												
λ = 0.95	0.13	45.1	1.00	53.4	0.26	43.4	0.33	38.4	0.40	39.4	1.00	15.2
* P* = 5%	0.15	32.3	1.00	45.7	0.09	47.7	0.21	45.2	0.32	48.3	1.00	19.9
*f*_6_												
λ = 0.95	0.72	95.1	0.87	54.2	0.72	82.4	0.88	71.2	0.78	72.3	1.00	33.6
*P* = 5%	0.81	30.2	0.91	24.3	0.56	41.9	0.67	41.8	0.62	47.7	1.00	18.3

In these simulations, the transition probability matrices of the Markov chain models were updated and the corresponding stationary distributions of the states change accordingly. As shown in Fig. 2p–r, the stationary distributions $\pi = \left( {\pi_{1} ,\pi_{2} , \ldots ,\pi_{N} } \right)$ change gradually with the update of transition probability matrices and are finally convergent. Finally, the shape of the stationary distribution resembles the shape of the drug response function, which explains why the algorithm we proposed is effective for searching for the optimal combinatorial drugs.

### Predicting the optimal combinatorial concentrations of multiple drugs

Simulation experiments were also conducted to validate the Markov chain-based algorithm and to compare its performance with the benchmark algorithms in the case of two drugs and three drugs respectively. The two response functions of two drugs are a Rastrigin-based function and a De Jong-based function, which are defined as below respectively:

$$\begin{gathered} f_{4} (x,y) = 333(0.475 - 0.005(x^{2} + y^{2} - 18\cos (2\pi x) - 10\cos (2\pi y))), \, where\, x,y \in [0,5]; \hfill \\ f_{5} (x,y) = 506( - 0.0029((x^{2} + y^{2} + 5\sin x^{2} + \cos y) + 0.015) + 0.0005), \, where \, x,y \in [0,5]. \hfill \\ \end{gathered}$$

In order to implement the simulation with $f_{4}$ and $f_{5}$, the entire range $[0,5]$, for $x$ and $y$ were evenly discretized into 20 distinct values.

The response function of three drugs, a ternary function, is defined as below:

$$f_{6} (x,y,z) = x^{2} + 2y^{2} - x^{2} y^{2} - z(x^{2} + y^{2} - 4),\;\; (x \ge 0,y \ge 0,z \ge 0).$$

This function has multiple stagnation points and a single maximum point. The process to analytical obtain the extreme value and the maximum value points is placed in the additional file 1. 100 pairs of $(x,y,z)$ including the extreme value and maximum points were randomly selected to represent different combinatorial drug concentrations and the drug response function values at these corresponding points were calculated. Then five searching algorithms are used to search for the maximum point.

**Fig. 3:**
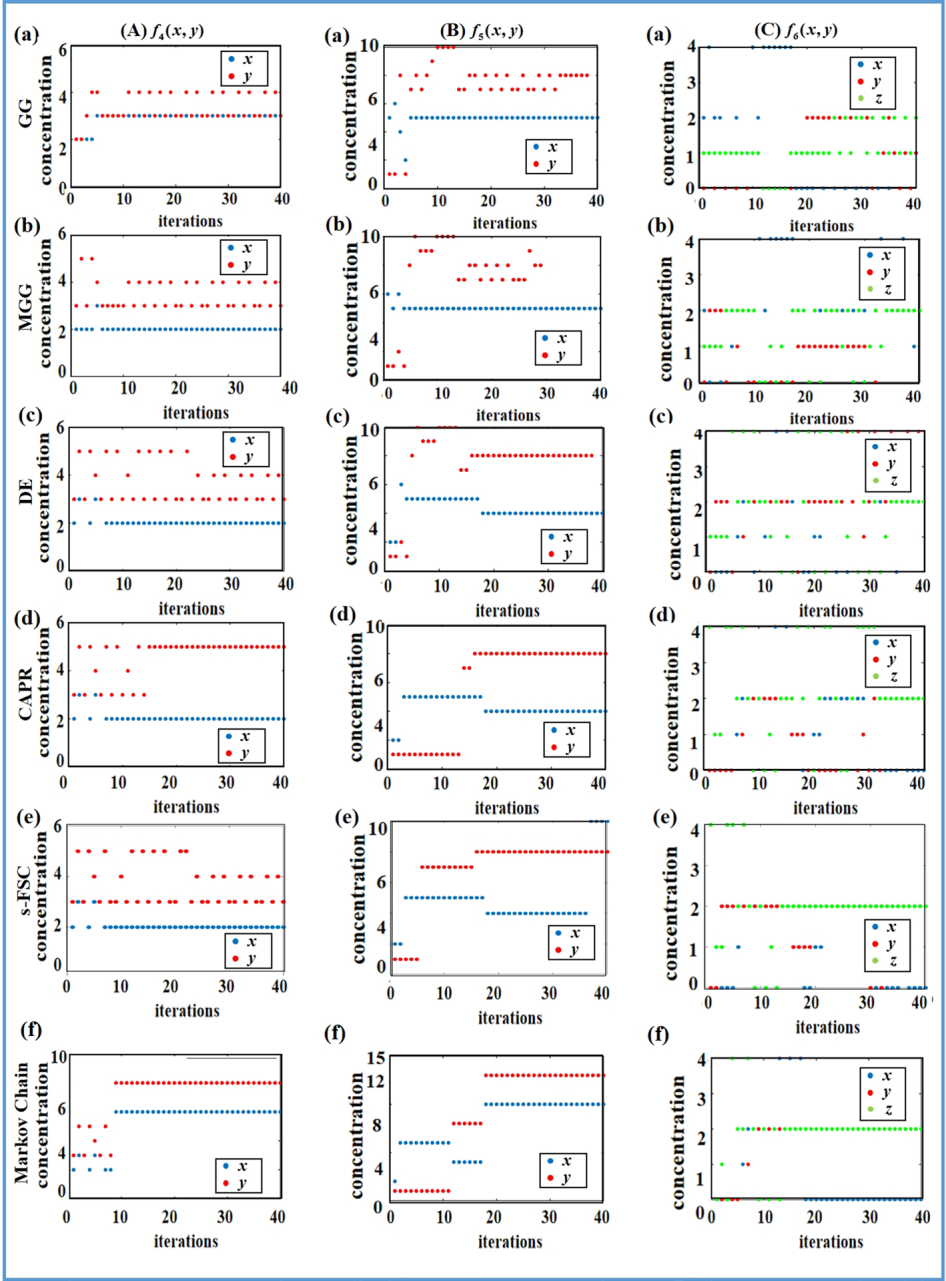
Numerical simulations of the response functions of multiple drugs using five different algorithms: **A** search process of the response function of two drug, $f_{4}$, using (*a*) GG algorithm; (*b*) MGG algorithm; (*c*) DE algorithm; (*d*) CAPR algorithm; (*e*) s-FSC algorithm; (*f*) Markov chain-based algorithm respectively; **B** search process of the response function of two drugs, $f_{5}$, using (*a*) GG algorithm; (*b*) MGG algorithm; (*c*) DE algorithm; (*d*) CAPR algorithm; (*e*) s−FSC algorithm; (*f*) Markov chain−based algorithm respectively; **C** search process of the response function, $f_{6}$^,^ using (*a*) GG algorithm; (*b*) MGG algorithm; (*c*) DE algorithm; (*d*) CAPR algorithm; (*e*) s−FSC algorithm; (*f*) Markov chain−based algorithm respectively

The searching processes of the optimal drug concentrations in the case of multiple drugs with the Markov chain-based algorithm and the five benchmark algorithms are displayed in Fig. 3. Figure 3A(a)–(f) in the left column, Fig. 3B(a)–(f) in the middle column, and Fig. 3C (a) ~ (f) in the right column of Fig. 3 represent the search for the optimal solutions of the response functions $f_{4}$, $f_{5}$ and $f_{6}$, respectively with the various algorithms, indicating the relationship between the number of iteration and the optimal solutions at each iteration. Each row of Fig. 3 represent the search for the optimal solutions of the three response functions of multiple drugs with a certain algorithm, and from the top to the bottom are the original GG algorithm, the MGG algorithm, the DE algorithm, the CAPR algorithm, the s-FSC algorithm, and the Markov chain-based algorithm respectively. From Fig. 3, the same conclusion can be drawn as the case of single drug that the five benchmark algorithms can easily fall into local optimal values or may take more iterations to find the optimal value, while the proposed Markov chain-based algorithm can find the global optimal value more reliably, but with less iterations.

**Fig. 4:**
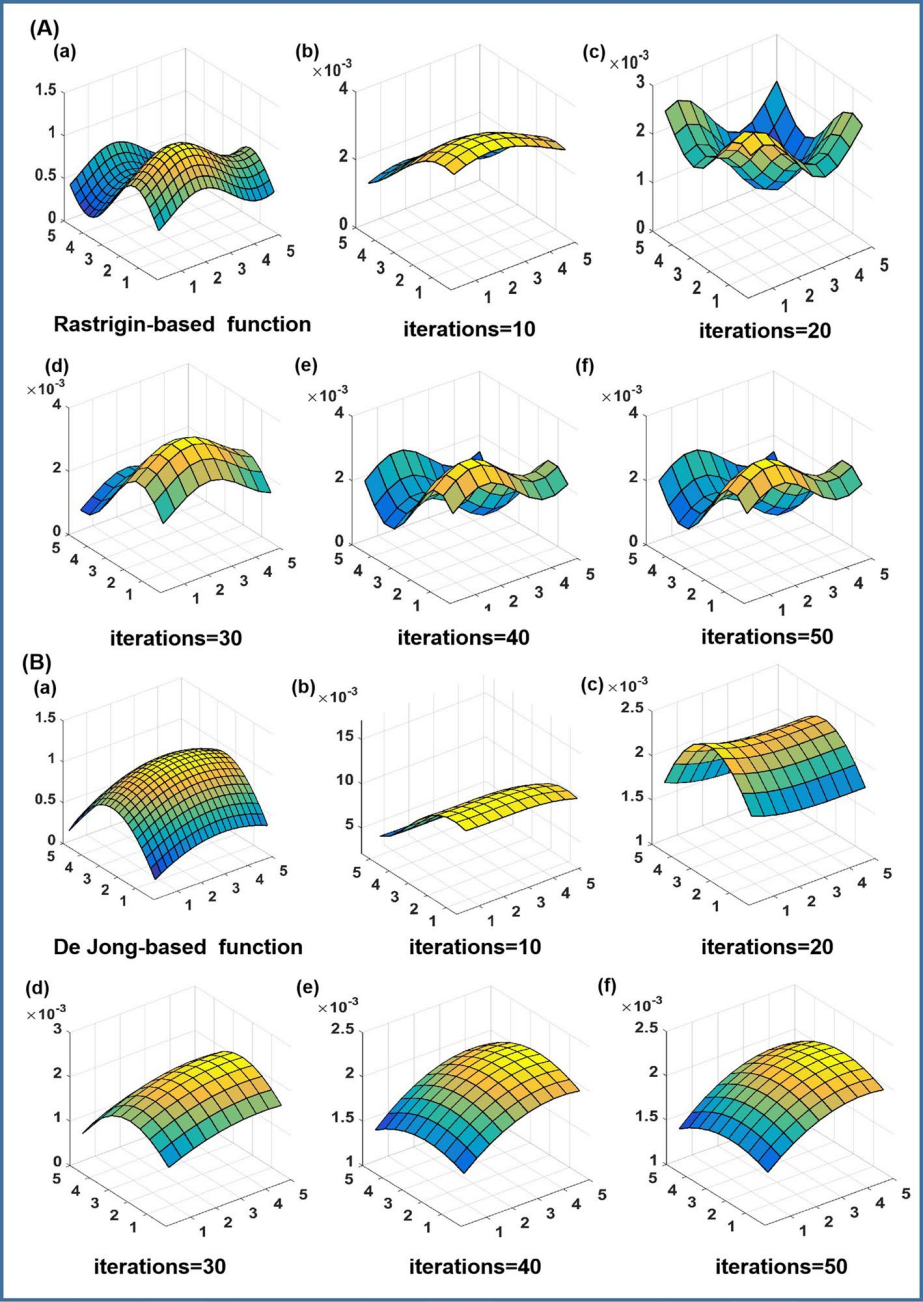
Two response functions of two drugs and corresponding stationary distributions at different iterations based on the Markov chain-based approach: as the number of iteration steps increases, the steady distribution surface converges to a surface pattern similar to the drug response surface: **A** Rastrigin-based function and **B** De Jong-based function

As stated in the section of Method Description, the essence of the optimization process of the Markov chain-based approach is to update the matrix of transition probability so that the corresponding stationary distribution of the states converges to a pattern, which has a similar shape to the drug responses. As shown in Fig. 4A(b)–(f) and B(b)–(f), the patterns of stationary distribution of the Markov chain models for the response functions of two drugs are displayed at different iterations, and the number of interval iterations between each graph is 10 steps. Figure 4A(a) and B(a) are the patterns of the response functions, the Rastrigin-based function and the De Jong-based function,respectively As the number of iterations increases, the smooth surfaces of the stationary distributions gradually converge in shape to the patterns of the corresponding response functions of two drugs.

Based on the results of the simulation experiments, the performances of the Markov chain-based algorithm and the other five algorithms were evaluated and compared with the measurements of success rate and number of iteration. The success rate of a algorithm indicates the effectiveness and reliability of the algorithm to find the optimal solutions, and the number of iterations represents how fast and efficient the algorithm is. The same evaluation measurements have been used to compare algorithm performance in literature [20], and they can effectively evaluate the reliability and efficiency of an algorithm.As shown in Table 1, the performance comparisons of the algorithms were made in the cases of the drug response functions of single drug ($f_{1}$**,** $f_{2}$ $f_{3}$), two drugs ($f_{4}$**,** $f_{5}$), and three drugs ($f_{6}$), respectively. The table lists success rate and searching iterations (# of iters) of each algorithm for every response function. For each function, 1000 simulation experiments were conducted. In each experiment, it is recorded as “success” if the maximum point was successfully found. The number of iterations at which the maximum point was found is defined as the iteration number of the experiment. The total number of successes divided by 1000 is the success rate of the algorithm, and the average number of iterations to find the maximum point is the searching iterations. The algorithm is regarded as effective if the optimized output is larger than the threshold (${\uplambda } = 0.95$) or the output we predicted is among the top $P = 5\%$ (even if the results we predicted is far from the real maximum value). From Table 1 we found that the success rate of the proposed Markov chain-based algorithm is much higher than the other benchmark algorithms, and the number of iterations is also less than the others. Furthermore, the success rate of the Markov chain-based algorithm is 1 in all cases of simulations, indicating that the Markov chain-based algorithm can always achieve the optimal solutions in the simulation experiments. Thus, it can be concluded that the reliability and efficiency of the Markov chain-based algorithm we proposed are better than the five benchmark algorithms.

Unlike of the benchmark algorithms of GG, MGG, DE, CAPR and s-FSC depicted in Additional file 1, the Markov chain-based algorithm can surmount the deficiencies that the benchmark methods have, and can always successfully predict the optimal concentrations of combinatorial drugs with excellent performance in all simulation experiments (Figs. 2, 3 and Table 1). The the state space of Markov chain models are constructed by discetizing the drug concentrations evenly and the experimental points can be selected randomly. The state with the largest steady-state probability in the stationary distribution is the output of the Markov chain-based method and such output is usually unique. Moreover, in the simulations, with the benchmark algorithms of GG, MGG, DE CAPR, s-FSC in the FSC framework, the prediction of combinatorial drug concentrations at a certain iteration is conducted based on the drug response information at the previous iteration, and then the experiments with predicted drug concentrations are carried out for the next iteration of prediction. On the contrary, the Markov chain-based approach allows the experiments at the selected states or drug concentrations to be implemented simultaneously. This makes a significant difference for biologically optimizing the combinatorial drug concentrations since it usually takes a few hours or days to implement one iteration of biological experiments and the computational time is usually much less than the time spent in the biological experiments. Therefore, it make take more than a month to finish a concentration optimization of combinatorial drugs using the benchmark algorithms in the FSC framework. However, the Markov chain-based approach with parallel experiments at the selected set of drug concentrations could save lots of time in the biological experiments. From this perspective, the Markov chain-based algorithm is much more efficient than the benchmark algorithms.

## Biological experiments and discussion

### Cell culture

The cell lines used in this study were obtained from the School of Medical Device, Shenyang Pharmaceutical University (Shenyang, China). MCF-7 cells (human breast cancer cell line) and BXPC-3 cells (human pancreatic cancer cell line) were cultured in RPMI-1640 (Thermo Scientific HyClone, Logan, UT, USA) containing 10% fetal bovine serum and 1% penicillin–streptomycin solution at 37 °C (5% CO_2_).

### Cell proliferation assay

Cells were plated onto 96-well plates (8 × 10^3^ cells/well for MCF-7 and 8 × 10^3^ cells/well for BXPC-3) and allowed to attach for 24 h. Cells were incubated with free drugs dissolved in an appropriate cell culture medium at serial concentrations for 72 h. For treatments containing DOX and PTX, each contained nine concentrations ranging from 0 to 5000 nM according to DOX-equivalent concentration with a total of 81 concentration combinations with six complex holes per concentration. Following incubation, 10 µL of cell counting kit-8 (CCK8) (Dojindo) was added to each well in the dark and incubated at 37 °C (5% CO_2_) for 2 h. After incubation, a microplate reader (Thermo, Multiskan FC) was used to measure the number of viable cells in each well of a 96-well plate at a wavelength of 450 nm.

### Performance comparison

**Fig. 5:**
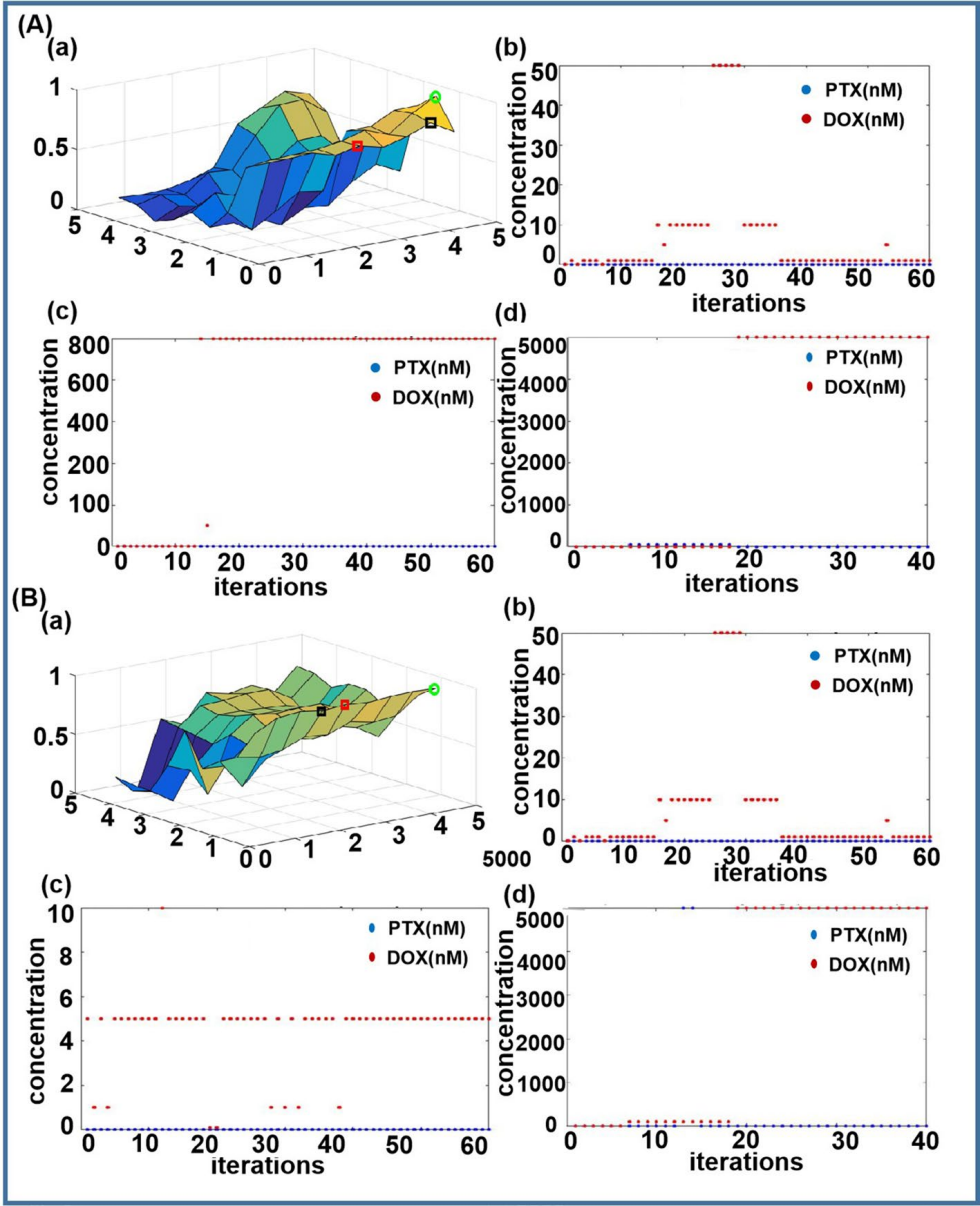
Two drug response functions and numerical simulations using three different algorithms: **A** drug response function of MCF-7 cells to PTX and DOX. (*a*) drug response function; (*b*) using GG algorithm; (*c*) using MGG algorithms; (*d*) using Markov chain-based algorithm; **B** drug response function of BXPC-3 cells to PTX and DOX: (*a*) drug response function; (*b*) using GG algorithm; (*c*) using MGG algorithms (*d*) using Markov chain-based algorithm

The response functions of the cells, MCF-7 and BXPC-3, under the combinatorial action of two drugs, paclitaxel (PTX) and doxorubicin hydrochloride (DOX), were constructed based on the biological responses to compare the performance of the algorithm we proposed and the GG and MGG algorithms. Figure 5A-(a) and B-(a) are the two drug response functions. (The green circle drawn in the fig is the maximum point of the drug response function, the red square is the maximum point found using the GG algorithm, and the black square is the maximum point found using the MGG algorithm.) Fig. 5A-(b) and B-(b) are the performance of the original GG algorithm, Fig. 5A-(c) and B-(c) are the performance of the MGG algorithm, and Fig. 5A-(d) and B-(d) are the performance of the Markov chain-based algorithm to find the optimal combination of PTX and DOX. Figure 5A(b)–(c) and B(b)–(c) show the nonrobustness of the GG and MGG algorithms. From Fig. 5, we can draw conclusions similar to those in the simulation. The original GG algorithm can easily lead to falling into the local optimal value (Fig. 5A(b) and B(b)). As shown in Fig. 5A(c) and B(c), the MGG algorithm may take many iterations if the starting point and the optimal state are far away. It is obvious that the point found by MGG algorithm is the local optimal value as shown in the black square in Fig. 5A(a) and B(a).

In the Fig. 5A(d) and B(d), when the Markov chain-based algorithm is used, the global optimal combination can be found within only a few iterations. As the experiment and calculation are parallel, the proposed algorithm is much more efficient.

Shown in Table 2 are performance comparisons of the two GG-based algorithms and the Markov chain-based algorithm. Similar to the results of simulation, the efficiency and accuracy of the Markov chain-based algorithm are much better than those of the GG and MGG stochastic algorithms.

**Table 2:** Performance comparison of three algorithms in biological experiments

	GG algorithm		MGG algorithm		Markov Chain algorithm	
	Success rate	# of iters	Success rate	# of iters	Success rate	# of iters
MCF-7						
${\uplambda } = 0.7$	0.20	10.8	0.39	5.4	1.00	19.0
${\uplambda } = 0.8$	0	NaN	0	NaN	1.00	19.0
${\uplambda } = 0.9$	0	NaN	0	NaN	1.00	19.2
$P = 5\%$	0	NaN	0	NaN	1.00	19.2
BXPC-3						
${\uplambda } = 0.7$	0.48	11.0	0.20	1.0	1.00	7.0
${\uplambda } = 0.8$	0.05	8.2	0	NaN	1.00	7.0
${\uplambda } = 0.9$	0	NaN	0	NaN	1.00	19.0
$P = 5\%$	0	NaN	0	NaN	1.00	19.0

**Fig. 6:**
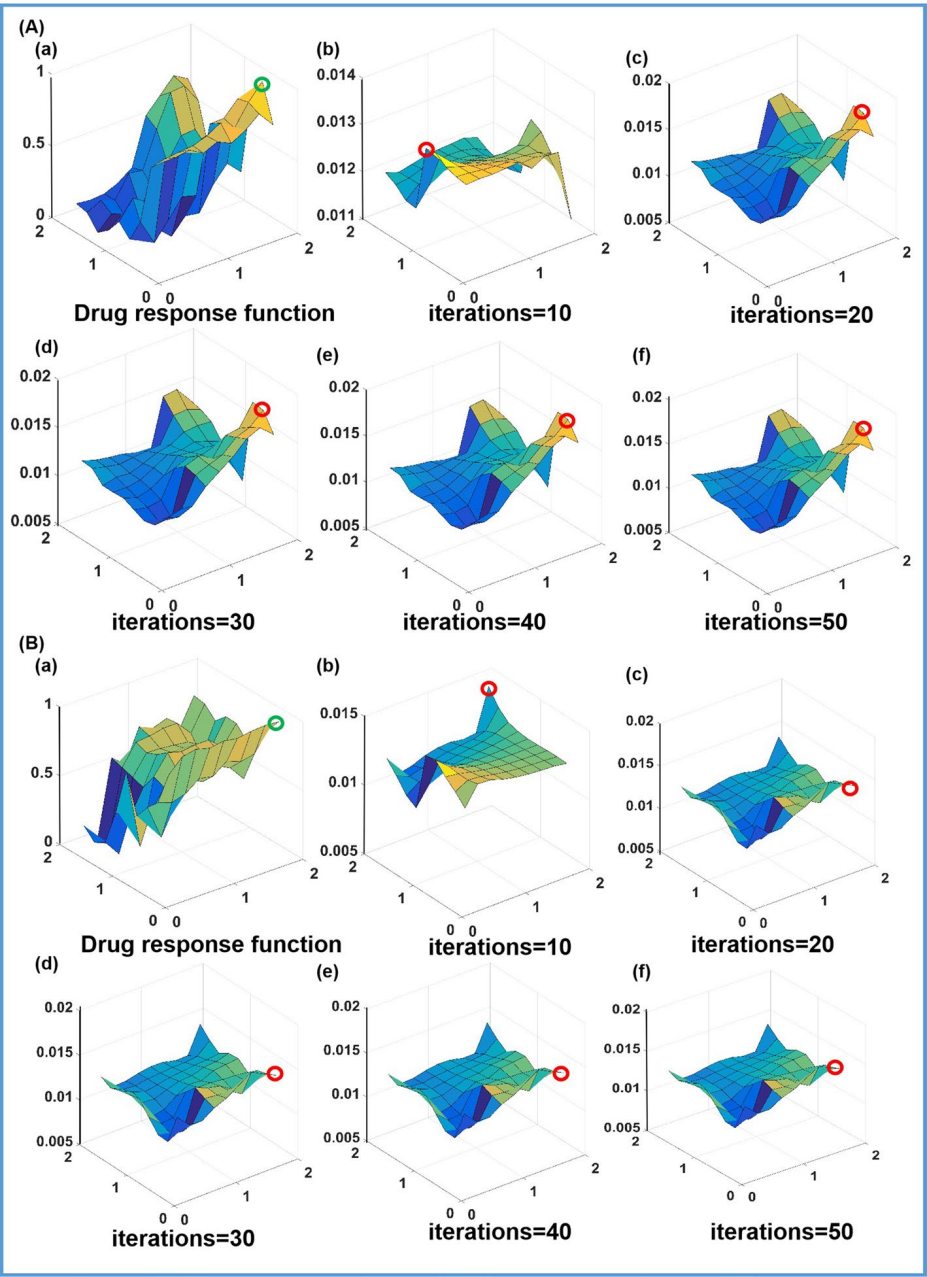
Stationary distributions of the states of the Markov chain models are convergent to the shapes similar to the drug response functions as the transition probability matrices are updated, and the optimal combinatorial drug concentrations correspond to the maximum points on the convergent pattern of the stationary distribution

As shown in Fig. 6, the stationary distribution of the states (the combinatorial drug concentrations) of the Markov chain model are convergent to the drug response functions of the cells. Figure 6A(a) and B (a) are the two response functions of the cell lines, MCF-7 and BXPC-3, under the action of the combinatorial drugs respectively (the green circle is the maximum value of the drug response function). Figure 6A(b)–(f) and B(b)–(f) are the stationary distribution at the selected iterations. (The red circle is the maximum point found at the corresponding iteration.) The number of interval iterations between each graph is 10 steps. It can be concluded that the stationary distributions of the combinatorial drug concentrations varied as the transition probability matrices were updated. Finally, the pattern shapes of the stationary distribution is convergent and similar to the corresponding drug response functions. At approximately 20 iterations, the optimal drug concentrations can be found, which explains why the Markov chain-based algorithm performs very well for optimizing the combinatorial drug concentrations.

## Conclusion

In this study, a novel Markov chain-based approach was proposed to solve the problem of the concentration optimization of combinatorial drugs. Its basic principle was introduced and the detailed algorithm was depicted in a general case. Both simulation and biological experiments were implemented to validate the proposed approach and to compare its performance with five benchmark algorithms, GG, MGG, DE, CAPR and s-FSC, in a FSC framework. The simulation experiments were conducted with the response functions of single drug, two drugs, and three drugs, and the biological experiments were carried out in the case of two drugs with two types of cells, respectively The performances of the Markov chain-based approach and the benchmark algorithms were evaluated using two measurements, the success rate and the number of iteration, indicating the reliability and efficiency of a algorithm to seek the global optimum. The experimental results and the comparisons between the proposed method and the benchmark algorithms demonstrate that the Markov chain-based approach is much more reliable and efficient than the selected benchmark algorithms in the FSC framework. The Markov chain-based algorithms can always succeed in achieving the optimal solutions with much less computational iterations. Moreover, considering that the time spent in the biological experiments is much more than the computational time, the Markov chain-based approach allows parallel experiments at the selected set of drug concentrations and could save lots of time in the biological experiments, and therefore the proposed method will be much more efficient than the benchmark algorithms in the practical application.

## Supplementary Information

**Additional file 1.** Theory of Markov chain-based method and other benchmark algorithms.

**Additional file 2**. Figure S1. State-transition diagram of an Markov chain.

**Additional file 3**. Figure S2. Drug response function between two adjacent concentrations.

**Additional file 4**. Figure S3. Initializing the Markov chain and updating the corresponding transition probability according to two adjacent experimental points.

**Additional file 5**. Figure S4. Two-drug case: a two-dimensional network structure with N^2^  states.

**Additional file 6**. Figure S5. Drug response function at concentration levels (x,y) and (x,y+1).

**Additional file 7**. Figure S6. Initializing the Markov chain and updating the corresponding Markov-chain-based transition probability based on the two neighboring states. 

## Data Availability

The datasets used and/or analysed during the current study available from the corresponding author on reasonable request.
