# Supplementary material for: Concentration optimization of combinatorial drugs using Markov chain-based models
Source: BMC Bioinformatics. 2021 Sep 21;22:451. doi: 10.1186/s12859-021-04364-5 (PMC8456646; doi:10.1186/s12859-021-04364-5)
Supplement: Supplementary file 1 — Additional file 1. Theory of Markov chain-based method and other benchmark algorithms. [file 12859_2021_4364_MOESM1_ESM.pdf]

Experimental Data:

```
data1 = [
    -0.66   -0.13   2.48    6.83    27.11   33.45   39.93   36.52   -1.01   2.10
    -0.05   9.41    8.49    24.08   37.81   38.43   29.84   -6.76   -0.95   -4.57
    -1.52   2.75    12.45   22.03   22.51   11.33 -3.01     1.79    -1.32   0.20
    5.94    10.96   16.05   18.47   8.45    3.59    11.53   7.75    1.82    13.23
    12.50   26.49   27.91   14.13   -1.31   -1.13   -1.17   -6.50   0.19
    -0.80   9.31    16.25   10.48   9.04    -2.06   5.50    13.53   11.75
    40.50   40.72   46.99   47.31 35.29     36.96   37.61   38.23   40.29   43.81
    39.36   48.32   55.05   40.26   40.41   41.38   37.94   40.66   37.81
    41.17   46.09   39.29
    ];

data2 = [
    0.00 -11.65     40.82   33.13   55.70   51.49   54.97   45.44   64.08   -7.27
    -11.23  38.48   52.82   53.82   55.94   56.01   45.67   62.69 -2.62     -14.14
    36.06   49.80   49.49   48.47   47.21   33.44   55.32   22.37   15.37   33.37
    53.13   51.29   50.74   50.20   45.77   62.64 55.69     -0.17   45.42   58.68
    56.14   57.48   57.46   49.23   62.28 29.03     27.76   42.02   49.52   51.27
    54.86   52.73   47.87   53.94 39.37     15.13   34.06   49.16   43.85   51.52
    46.85   35.77   58.64 47.08     43.79   46.60   53.81   52.09   59.31   55.53
    57.53   71.36 82.18     81.30   81.77   79.58   81.12   79.59   81.22   81.88
    81.14
    ];
```

GG algorithm

```
clear
clc
close all
%% data1 = [
	-0.66	-0.13	2.48	6.83	27.11	33.45	39.93	36.52	-1.01	2.10
	-0.05	9.41	8.49	24.08	37.81	38.43	29.84	-6.76	-0.95	-4.57
	-1.52	2.75	12.45	22.03	22.51	11.33 -3.01	1.79	-1.32	0.20
	5.94	10.96	16.05	18.47	8.45	3.59	11.53	7.75	1.82	13.23
	12.50	26.49	27.91	14.13	-1.31	-1.13	-1.17	-6.50	0.19
	-0.80	9.31	16.25	10.48	9.04	-2.06	5.50	13.53	11.75
	40.50	40.72	46.99	47.31 35.29	36.96	37.61	38.23	40.29	43.81
	39.36	48.32	55.05	40.26	40.41	41.38	37.94	40.66	37.81
	41.17	46.09	39.29
	];

data2 = [
	0.00 -11.65	40.82	33.13	55.70	51.49	54.97	45.44	64.08	-7.27
	-11.23	38.48	52.82	53.82	55.94	56.01	45.67	62.69 -2.62	-14.14
	36.06	49.80	49.49	48.47	47.21	33.44	55.32	22.37	15.37	33.37
	53.13	51.29	50.74	50.20	45.77	62.64 55.69	-0.17	45.42	58.68
	56.14	57.48	57.46	49.23	62.28 29.03	27.76	42.02	49.52	51.27
	54.86	52.73	47.87	53.94 39.37	15.13	34.06	49.16	43.85	51.52
	46.85	35.77	58.64 47.08	43.79	46.60	53.81	52.09	59.31	55.53
	57.53	71.36 82.18	81.30	81.77	79.58	81.12	79.59	81.22	81.88
	81.14
	];
con_x=[0 0.1 1 5 10 50 100 1000 5000];
con_y=[0 0.1 1 5 10 50 100 1000 5000];
data = data2;
data_std = (data-min(min(data)))/(max(max(data))-min(min(data)));
num_col = size(data1,2);
  for i=1:num_col
        if(i<=40)
            state(i)= (fix(num_col/2))*(-1);
            num_col=num_col-2;
        elseif (i>40 && i<=80)
                state(i)=i-40;
        end
  end

for i=1:1:81
    stop(i)=0;
end
```

```
for i=1:1:60
        response(i)=0;
end
for i=1:1:60
        hang(i)=0;
        lie(i)=0;
end

a=0;
j=1;
i=1;
iteration=0;
count2=0;
max=stop(1);
while(j>0&&iteration<60)
      a=unifrnd(0,1);
      if ((data_std(j)<a)&(state(j)<0))          % state(i)<0 penalty
              j=j+1;
      elseif ((data_std(j)<a)&(state(j)>0))          % state(i)>0 penalty
              j=j-1;
      elseif ((data_std(j)>=a)&(state(j)==40))|((data_std(j)>=a)&(state(j)==-40))
              j=j;
      elseif ((data_std(j)>=a)&(state(j)<0))
              j=j-1;
      elseif ((data_std(j)>=a)&(state(j)>0))
              j=j+1;
      end
      iteration=iteration+1;
      stop(j)=stop(j)+1;
      while(i<=iteration)
                      if (stop(j)>=max)
                              max = stop(j);
                              markx=j;
                      end
                      response(i)=data_std(markx);
                      concentration_x = ceil(markx/9);
                      if mod(markx,9)~=0
                            concentration_y = mod(markx,9);
                      else
                              concentration_y=9;
                      end
                      hang(i)=con_x(concentration_x);
                      lie(i)=con_y(concentration_y);
                      i=i+1;
```

```
    end
    if (iteration>=60)
        break;
    end
end
state;
plot(hang,'.b');
hold on;
plot(lie,'.r');
xlabel('iteration');
ylabel('concentration');
legend('PTX-concentration（nM）','DOX-concentration（nM）');
title('Optimize Combinatory Drugs(Gurgame)')
figure(2);
plot(response,'.');
%}
```

MGG algorithm

```
clear
clc
close all
%% data1 = [
	-0.66	-0.13	2.48	6.83	27.11	33.45	39.93	36.52	-1.01	2.10
	-0.05	9.41	8.49	24.08	37.81	38.43	29.84	-6.76	-0.95	-4.57
	-1.52	2.75	12.45	22.03	22.51	11.33 -3.01	1.79	-1.32	0.20
	5.94	10.96	16.05	18.47	8.45	3.59	11.53	7.75	1.82	13.23
	12.50	26.49	27.91	14.13	-1.31	-1.13	-1.17	-6.50	0.19
	-0.80	9.31	16.25	10.48	9.04	-2.06	5.50	13.53	11.75
	40.50	40.72	46.99	47.31 35.29	36.96	37.61	38.23	40.29	43.81
	39.36	48.32	55.05	40.26	40.41	41.38	37.94	40.66	37.81
	41.17	46.09	39.29
	];

data2 = [
	-11.65	40.82	33.13	55.70	51.49	54.97	45.44	64.08	-7.27	-11.23
	38.48	52.82	53.82	55.94	56.01	45.67	62.69 -2.62	-14.14	36.06
	49.80	49.49	48.47	47.21	33.44	55.32 22.37	15.37	33.37	53.13
	51.29	50.74	50.20	45.77	62.64 55.69	-0.17	45.42	58.68	56.14
	57.48	57.46	49.23	62.28	29.03	27.76	42.02	49.52	51.27	54.86
	52.73	47.87	53.94 39.37	15.13	34.06	49.16	43.85	51.52	46.85
	35.77	58.64 47.08	43.79	46.60	53.81	52.09	59.31	55.53	57.53
	71.36 82.18	81.30	81.77	79.58	81.12	79.59	81.22	81.88	81.14
	];

data = data2;
data_std = (data-min(min(data)))/(max(max(data))-min(min(data)));
num_col = size(data_std,2);
for i=1:1:1000
    suc(i)=0;
    iter(i)=0;
end
success=0;
for p=1:1:80
   stop(p)=0;
end
for q=1:1:60
	response(q)=0;
end

for repeat=1:1:1000
a=0;
```

```
k=3;
i=1;
iteration=0;
count2=0;
max=stop(1);
while(k>=0&&iteration<60)
    a=unifrnd(0,1);
      if(data_std(k-1)<data_std(k))&((ga(data_std(k-1),data_std(k))<a)&(k>2))
          k=k-1;
    elseif(data_std(k-1)<data_std(k))&((ga(data_std(k-1),data_std(k))>=a)&(k<num_col-1))
          k=k+1;
    elseif (data_std(k)>data_std(k+1))&((ga(data_std(k),data_std(k+1))>=a)&(k<num_col-1))
          k=k+1;
    elseif (data_std(k)>data_std(k+1))&((ga(data_std(k),data_std(k+1))<a)&(k>2))
          k=k-1;
    elseif(data_std(k-1)>data_std(k))&((ga(data_std(k-1),data_std(k))>=a)&(k>2))
          k=k-1;
    elseif(data_std(k-1)>data_std(k))&((ga(data_std(k-1),data_std(k))<a)&(k<num_col-1))
          k=k+1;
    elseif(data_std(k+1)<data_std(k))&((ga(data_std(k+1),data_std(k))<a)&(k<num_col)-1)
          k=k+1;
    elseif(data_std(k+1)<data_std(k))&((ga(data_std(k+1),data_std(k))>=a)&(k>2))
          k=k-1;
    else k=k;
  end
    stop(k)=stop(k)+1;
    iteration=iteration+1;
        while(i<=iteration)
                if (stop(k)>=max)
                      max = stop(k);
                      markx=k;
                end
                response(i)=data_std(markx);
                i=i+1;
    end
    response(iteration)=data_std(markx);
    if ((iteration<60)&(response(iteration)>=0.))
          suc(repeat)=1;
          success=success+1;
          iter(repeat)=iteration;
          break;
    elseif (iteration>=60)
          suc(repeat)=0;
          iter(repeat)=0;
```

```
            break;
        end
    end
end
sum_suc=0;
iter_sum=0;
iter_average=0;
suc_average=0;
for i=1:1:1000
    sum_suc=sum_suc+suc(i);
    if(suc(i)==1)
        iter_sum=iter_sum+iter(i);
    end
end
    iter_average=iter_sum/success;
    suc_average=sum_suc/1000;

iter_average
suc_average
success
```

DE algorithm

```
function DE(Gm,F0)
Gm=40;
Np=100;
CR=0.9;
G=1;
N=10;
ge=zeros(1,Np);
bestx=zeros(Np,N);

%xmin=-10;xmax=100;
xmin=-5.12;xmax=5.12;
function y=f(XX)
    y=sum(XX.^2-10.*cos(2.*pi.*XX)+10);
end
X0=(xmax-xmin)*rand(Np,N)+xmin;
X=X0;

X1new=zeros(Np,N);
X1_new=zeros(Np,N);
X1=zeros(Np,N);
value=zeros(1,Np);
while G<=Gm
    for i=1:Np

    a=1;b=Np;
    dx=randperm(b-a+1)+a-1;
    j=dx(1);k=dx(2);p=dx(3);
    if j==i
        j=dx(4);
    elseif k==i
        k=dx(4);
    elseif p==i
        p=dx(4);
    end
    namd=exp(1-Gm/(Gm+1-G));
    F=F0*2.^namd;
    bon=X(p,:)+F*(X(j,:)-X(k,:));
    if (bon>xmin)&(bon<xmax)
       X1new(i,:)=bon;
    else X1new(i,:)=(xmax-xmin)*rand(1,N)+xmin;
    end
    end
```

```
        for i=1:Np
            if rand>CR
                X1_new(i,:)=X(i,:);
            else
                X1_new(i,:)=X1new(i,:);
            end
        end

   for i=1:Np
       if f(X1_new(i,:))<f(X(i,:))
           X1(i,:)=X1_new(i,:);
       else
           X1(i,:)=X(i,:);
       end
   end

         for i=1:Np
              value(i)=f(X1(i,:));
         end
        [fmin,nmin]=min(value);
        ge(G)=fmin;
        bestx(G,:)=X1(nmin,:);
        G=G+1;
        X=X1;
end
ii=linspace(1,N,Np);
plot(ii,ge)
[gmin,n]=min(ge);
bestvalue=gmin
bestsolution=bestx(n,:)

end
```

CAPR algorithm

```
xmin=0;xmax=28;
G=1;
Gm=41;
Np=28;
CR=0.5;
rmin=0.1;rmax=1;wmax=0.2; wmin=0;M=4;
F=0.5;
D=1;
X0=zeros(Np,D);
oX0=zeros(Np,D);
X1=zeros(Np,D);
u=zeros(Np,D);
X2=zeros(Np,D);
bestx=zeros(40,D);
value2=zeros(Np,D);
value=zeros(Np,D);

% X0=randi(13,Np,D);
r1=rmax - G*(rmax-rmin)/Gm;
r2=wmin + G*(wmax-wmin)/Gm;

%oX0= xmin + xmax - x0;
while ( G <Gm)
        X0=randi(25,Np,D);
  for i=1:Np
          value(i)=f3(X0(i,:));
  end
  [fmax,p]=max(value);
  dG=average(f3(X(G)));



  for i=1:Np
        a=1;b=Np;
        dx=randperm(b-a+1)+a-1;
        j=dx(1);k=dx(2);
        %mutation process
        rand_1=rand(1,1);
        if (rand_1<=r1)
              %X1(i,:) = X0(i,:) + F* (X0(j,:) - X0(k,:));
              bon= X0(i,:) + F* (X0(j,:) - X0(k,:));
        else
              %X1(i,:) = X0(p,:) + F* (X0(j,:) - X0(k,:));
              bon= X0(p,:) + F* (X0(j,:) - X0(k,:));
```

```
    end
      if (bon>xmin)&(bon<xmax)
            X1(i,:)=bon;
      else
            X1(i,:)=randi(25,1,D);
      end
end
for i=1:Np
    jrand= floor(D * rand_1) +1;

    for j=1:D
        if (rand_1 <= CR || j == jrand)
            u(i,j)=X1(i,j);
        else
            u(i,j)=X0(i,j);
        end
    end

    if(f3(u(i,:))> f3(X0(i,:)))
          X2(i,:)= u(i,:);
    else
          X2(i,:)=X0(i,:);
    end
    value2(i)=f3(X2(i,:));
end
    [fmax2,p2]=max(value2);
% Perturb the best individual dimension by dimension
    for j1=1:D
          k=floor(rand * Np) +1;
          n= floor(rand* D)+1;
          if (k==n)
                k=k+1;
          else
                k=k;
          end
          if (rand_1 <r2)
               bon1 = X0(p2,n) + (2*rand_1 -1)* (X0(p2,n)-X0(k,n));
          else
               bon1 = X0(p2,j1) + (2*rand_1 -1)* (X0(p2,n)-X0(k,n));
          end
          if (bon1>xmin)&(bon1<xmax)
            uu(p2,j1)=bon1;
          else
            uu(p2,j1)=randi(25,1,D);
```

```
                end
                    value3(p2,j1)=f3(uu(p2,j1));
        end
      [fmax3,p3]=max(value3);
          if(fmax3 >= fmax2)
                ge(G)=fmax3;
                bestx(G,:)=uu(p3,:)+1;
          else
                ge(G)=fmax2
                bestx(G,:)=X2(p3,:)+1;
          end
    G=G+1;
end


plot(bestx,'- .');
axis([0,40,0,25]);
title('Drug Response');
xlabel('x');
ylabel('f(x)');
%    function y=f1(X)
%          y=(-1/72)*(X-6)*(X-6)+1;
%    end
%
%    function y=f2(X)
%          y=0.0033 * (X-14)*(X-14) +0.35;
%    end
    function y=f3(X)
          y= max ((-7)/640*(X-19)*(X-19) +0.8 ,(-1)/180*(X-6)*(X-6) +0.3) +0.2;
    end
%        %% De Jong's function1
%      function z=f4(X,Y)
%          z=    sum(X.^2 + Y.^2+ 5* sin(X.^2)* cos(X));
%      end
%      %% Ratrigin_fuc6
%      function z=f5(X,Y)
%              z=0.6 + 0.2/12*sum(X.^2+Y.^2-5*cos(2*pi*X)-3*cos(2*pi*Y) );
%      end
```

Markov chain based algorithm

```
clear
clc
close all
%% data1 = [
	0.00	-0.66	-0.13	2.48	6.83	27.11	33.45	39.93	36.52
	-1.01	2.10	-0.05	9.41	8.49	24.08	37.81	38.43	29.84
	-6.76	-0.95	-4.57	-1.52	2.75	12.45	22.03	22.51	11.33
	-3.01	1.79	-1.32	0.20	5.94	10.96	16.05	18.47	8.45
	3.59	11.53	7.75	1.82	13.23	12.50	26.49	27.91	14.13
	-1.31	-1.13	-1.17	-6.50	0.19	-0.80	9.31	16.25	10.48
	9.04	-2.06	5.50	13.53	11.75	40.50	40.72	46.99	47.31
	35.29	36.96	37.61	38.23	40.29	43.81	39.36	48.32	55.05
	40.26	40.41	41.38	37.94	40.66	37.81	41.17	46.09	39.29
	];


data2 = [
	0.00 -11.65	40.82	33.13	55.70	51.49	54.97	45.44	64.08
	-7.27	-11.23	38.48	52.82	53.82	55.94	56.01	45.67	62.69
	-2.62	-14.14	36.06	49.80	49.49	48.47	47.21	33.44	55.32
	22.37	15.37	33.37	53.13	51.29	50.74	50.20	45.77	62.64
	55.69	-0.17	45.42	58.68	56.14	57.48	57.46	49.23	62.28
	29.03	27.76	42.02	49.52	51.27	54.86	52.73	47.87	53.94
	39.37	15.13	34.06	49.16	43.85	51.52	46.85	35.77	58.64
	47.08	43.79	46.60	53.81	52.09	59.31	55.53	57.53	71.36
	70.77	80.30	82.18	79.58	81.12	79.59	81.22	81.88	81.14
	];

data = data2;
data_std = (data-min(min(data)))/(max(max(data))-min(min(data)));
num_row = size(data_std,1);
num_col = size(data_std,2);

x = 1:num_row;
y = 1:num_col;
y = num_col:-1:1;
z=1:num_row*num_col;
%{
x-con={'0','0.1','1','5','10','50','100','1000','5000'};
set(gca, 'Xtick',[0 1 2 3 4 5 6 7 8 ]);
set(gca, 'XtickLabel',{'0','0.1','1','5','10','50','100','1000','5000'});
set(gca, 'Ytick',[0 1 2 3 4 5 6 7 8 ]);
set(gca, 'YtickLabel',{'0','0.1','1','5','10','50','100','1000','5000'});
```

```
%}
surf(x,y,data_std)
con={'0','0.1','1','5','10','50','100','1000','5000'};
con1={'5000','1000','100','50','10','5','1','0.1','0'};
set(gca, 'XtickLabel',con);
set(gca, 'YtickLabel',con);
xlabel('DOX-concentration(nM)')
ylabel('PTX-concentration(nM)')
zlabel('f(x)')
title('Drug Response')
%}
%
% num_row = 20;
% num_col = 20;
%
num_elm = num_row*num_col;
P = zeros(num_elm,num_elm);
%
P(1,1) = 2/4;
P(1,2) = 1/4;
P(1,num_col+1) = 1/4;
P(num_col,num_col) = 2/4;
P(num_col,num_col-1) = 1/4;
P(num_col,num_col+num_col) = 1/4;
P((num_row-1)*num_col+1,(num_row-1)*num_col+1) = 2/4;
P((num_row-1)*num_col+1,(num_row-1)*num_col+2) = 1/4;
P((num_row-1)*num_col+1,(num_row-2)*num_col+1) = 1/4;
P(num_elm,num_elm) = 2/4;
P(num_elm,num_elm-1) = 1/4;
P(num_elm,num_elm-num_col) = 1/4;
%
for i = 1:num_elm
    if i > 1    &&    i < num_col
        P(i,i)=1/4;
        P(i,i-1) = 1/4;
        P(i,i+1) = 1/4;
        P(i,i+num_col) = 1/4;
    elseif i > (num_row-1)*num_col+1    &&    i < num_elm
        P(i,i)=1/4;
        P(i,i-1) = 1/4;
        P(i,i+1) = 1/4;
        P(i,i-num_col) = 1/4;
    elseif i ~= num_col && i ~= num_elm && mod(i,num_col) == 0
        P(i,i)=1/4;
```

```
            P(i,i-1)=1/4;
            P(i,i-num_col)=1/4;
            P(i,i+num_col)=1/4;
        elseif i ~= 1 && i ~= (num_row-1)*num_col+1 && mod(i,num_col) == 1
            P(i,i)=1/4;
            P(i,i+1)=1/4;
            P(i,i-num_col)=1/4;
            P(i,i+num_col)=1/4;
        elseif i > num_col+1 && i < (num_row-1)*num_col && mod(i,num_col) ~= 0 &&
mod(i,num_col) ~= 1
            P(i,i+1) = 1/4;
            P(i,i-1) = 1/4;
            P(i,i+num_col) = 1/4;
            P(i,i-num_col) = 1/4;
        end
end

pi = fun_staEqu(P);
pi_surf = reshape(pi,num_col,num_row)';
% figure
% surf(pi_surf)
% xlabel('x')
% ylabel('y')
% zlim([0,1])

num_smpPer =10 ;
%num_smpPer
=[1,2,7,8,9,10,19,20,21,22,23,24,25,26,27,28,29,30,31,32,33,34,35,36,37,38,39,40];
inter_row = floor(num_row/sqrt(num_smpPer/2));
inter_col = floor(num_col/sqrt(num_smpPer/2));

% num_smpPer = 9/81;
% disp(['Number of Samples: ',num2str(floor(num_elm*num_smpPer)),' / ',num2str(num_elm)])
% inter_row = floor(num_row/sqrt((num_elm*num_smpPer)/2));
% inter_col = floor(num_col/sqrt((num_elm*num_smpPer)/2));
idx = zeros(num_row,num_col);
for i = 1:inter_row:num_row
        for j = 1:inter_col:num_col
            idx(i,j) = 1;
        end
end
% idx

rand('seed',5)
```

```
[smp.row,smp.col] = find(idx == 1);
smp_len = length(smp.row);
rand_series = unifrnd(0,1,1,smp_len);

for i = 1:smp_len
        if smp.row(i) == 1 && smp.col(i) == 1
                if rand_series(i) > 0.5
                        epsilon = data_std(1,1) - data_std(1,2);
                        P(2,1) = P(2,1) + 0.25*epsilon;
                        P(2,2) = P(2,2) - 0.25*epsilon/3;
                        P(2,3) = P(2,3) - 0.25*epsilon/3;
                        P(2,2+num_col) = P(2,2+num_col) - 0.25*epsilon/3;

                        P(1,2) = P(1,2) - 0.25*epsilon;
                        P(1,1) = P(1,1) + 0.25*epsilon*2/3;
                        P(1,1+num_col) = P(1,1+num_col) + 0.25*epsilon/3;
                else
                        epsilon = data_std(1,1) - data_std(2,1);
                        P(1+num_col,1) = P(1+num_col,1) + 0.25*epsilon;
                        P(1+num_col,1+num_col) = P(1+num_col,1+num_col) - 0.25*epsilon/3;
                        P(1+num_col,1+num_col*2) = P(1+num_col,1+num_col*2) - 0.25*epsilon/3;
                        P(1+num_col,2+num_col) = P(1+num_col,2+num_col) - 0.25*epsilon/3;

                        P(1,1+num_col) = P(1,1+num_col) - 0.25*epsilon;
                        P(1,1) = P(1,1) + 0.25*epsilon*2/3;
                        P(1,2) = P(1,2) + 0.25*epsilon/3;
                end

        elseif smp.row(i) == 1 && smp.col(i) == num_col
                if rand_series(i) > 0.5
                        epsilon = data_std(1,num_col) - data_std(1,num_col-1);
                        P(num_col-1,num_col) = P(num_col-1,num_col) + 0.25*epsilon;
                        P(num_col-1,num_col-1) = P(num_col-1,num_col-1) - 0.25*epsilon/3;
                        P(num_col-1,num_col-2) = P(num_col-1,num_col-2) - 0.25*epsilon/3;
                        P(num_col-1,num_col+num_col-1)      =      P(num_col-1,num_col+num_col-1)      -
0.25*epsilon/3;

                        P(num_col,num_col-1) = P(num_col,num_col-1) - 0.25*epsilon;
                        P(num_col,num_col) = P(num_col,num_col) + 0.25*epsilon*2/3;
                        P(num_col,2*num_col) = P(num_col,2*num_col) + 0.25*epsilon/3;
                else
                        epsilon = data_std(1,num_col) - data_std(2,num_col);
                        P(num_col*2,num_col) = P(num_col*2,num_col) + 0.25*epsilon;
                        P(num_col*2,num_col*2) = P(num_col*2,num_col*2) - 0.25*epsilon/3;
```

```
                P(num_col*2,num_col*3) = P(num_col*2,num_col*3) - 0.25*epsilon/3;
                P(num_col*2,num_col*2-1) = P(num_col*2,num_col*2-1) - 0.25*epsilon/3;

                P(num_col,num_col*2) = P(num_col,num_col*2) - 0.25*epsilon;
                P(num_col,num_col) = P(num_col,num_col) + 0.25*epsilon*2/3;
                P(num_col,num_col-1) = P(num_col,num_col-1) + 0.25*epsilon/3;
            end

        elseif smp.row(i) == num_row && smp.col(i) == 1
            if rand_series(i) > 0.5
                epsilon = data_std(num_row,1) - data_std(num_row,2);
                P((num_row-1)*num_col+2,(num_row-1)*num_col+1) =
P((num_row-1)*num_col+2,(num_row-1)*num_col+1) + 0.25*epsilon;
                P((num_row-1)*num_col+2,(num_row-1)*num_col+2) =
P((num_row-1)*num_col+2,(num_row-1)*num_col+2) - 0.25*epsilon/3;
                P((num_row-1)*num_col+2,(num_row-1)*num_col+3) =
P((num_row-1)*num_col+2,(num_row-1)*num_col+3) - 0.25*epsilon/3;
                P((num_row-1)*num_col+2,(num_row-2)*num_col+2) =
P((num_row-1)*num_col+2,(num_row-2)*num_col+2) - 0.25*epsilon/3;

                P((num_row-1)*num_col+1,(num_row-1)*num_col+2) =
P((num_row-1)*num_col+1,(num_row-1)*num_col+2) - 0.25*epsilon;
                P((num_row-1)*num_col+1,(num_row-1)*num_col+1) =
P((num_row-1)*num_col+1,(num_row-1)*num_col+1) + 0.25*epsilon*2/3;
                P((num_row-1)*num_col+1,(num_row-2)*num_col+1) =
P((num_row-1)*num_col+1,(num_row-2)*num_col+1) + 0.25*epsilon/3;
            else
                epsilon = data_std(num_row,1) - data_std(num_row-1,1);
                P((num_row-2)*num_col+1,(num_row-1)*num_col+1) =
P((num_row-2)*num_col+1,(num_row-1)*num_col+1) + 0.25*epsilon;
                P((num_row-2)*num_col+1,(num_row-2)*num_col+1) =
P((num_row-2)*num_col+1,(num_row-2)*num_col+1) - 0.25*epsilon/3;
                P((num_row-2)*num_col+1,(num_row-2)*num_col+2) =
P((num_row-2)*num_col+1,(num_row-2)*num_col+2) - 0.25*epsilon/3;
                P((num_row-2)*num_col+1,(num_row-3)*num_col+1) =
P((num_row-2)*num_col+1,(num_row-3)*num_col+1) - 0.25*epsilon/3;

                P((num_row-1)*num_col+1,(num_row-2)*num_col+1) =
P((num_row-1)*num_col+1,(num_row-2)*num_col+1) - 0.25*epsilon;
                P((num_row-1)*num_col+1,(num_row-1)*num_col+1) =
P((num_row-1)*num_col+1,(num_row-1)*num_col+1) + 0.25*epsilon*2/3;
                P((num_row-1)*num_col+1,(num_row-2)*num_col+2) =
P((num_row-1)*num_col+1,(num_row-2)*num_col+2) + 0.25*epsilon/3;
            end
```

```
        elseif smp.row(i) == num_row && smp.col(i) == num_col
            if rand_series(i) > 0.5
                epsilon = data_std(num_row,num_col) - data_std(num_row,num_col-1);
                P(num_row*num_col-1,num_row*num_col)                                =
P(num_row*num_col-1,num_row*num_col) + 0.25*epsilon;
                P(num_row*num_col-1,num_row*num_col-1)                              =
P(num_row*num_col-1,num_row*num_col-1) - 0.25*epsilon/3;
                P(num_row*num_col-1,num_row*num_col-2)                              =
P(num_row*num_col-1,num_row*num_col-2) - 0.25*epsilon/3;
                P(num_row*num_col-1,num_col*(num_row-1)-1)                          =
P(num_row*num_col-1,num_col*(num_row-1)-1) - 0.25*epsilon/3;

                P(num_row*num_col,num_row*num_col-1)                                =
P(num_row*num_col,num_row*num_col-1) - 0.25*epsilon;
                P(num_row*num_col,num_row*num_col)                                  =
P(num_row*num_col,num_row*num_col) + 0.25*epsilon*2/3;
                P(num_row*num_col,num_col*(num_row-1))                              =
P(num_row*num_col,num_col*(num_row-1)) + 0.25*epsilon/3;
            else
                epsilon = data_std(num_row,num_col) - data_std(num_row-1,num_col);
                P(num_col*(num_row-1),num_col*num_row)                              =
P(num_col*(num_row-1),num_col*num_row) + 0.25*epsilon;
                P(num_col*(num_row-1),num_col*(num_row-1))                          =
P(num_col*(num_row-1),num_col*(num_row-1)) - 0.25*epsilon/3;
                P(num_col*(num_row-1),num_col*(num_row-1)-1)                        =
P(num_col*(num_row-1),num_col*(num_row-1)-1) - 0.25*epsilon/3;
                P(num_col*(num_row-1),num_col*(num_row-2))                          =
P(num_col*(num_row-1),num_col*(num_row-2)) - 0.25*epsilon/3;

                P(num_row*num_col,num_col*(num_row-1))                              =
P(num_row*num_col,num_col*(num_row-1)) - 0.25*epsilon;
                P(num_row*num_col,num_row*num_col)                                  =
P(num_row*num_col,num_row*num_col) + 0.25*epsilon*2/3;
                P(num_row*num_col,num_row*num_col-1)                                =
P(num_row*num_col,num_row*num_col-1) + 0.25*epsilon/3;
            end

        elseif smp.row(i) == 1 && smp.col(i) < num_col && smp.col(i) > 1
            if rand_series(i) < 1/3
                epsilon = data_std(1,smp.col(i)) - data_std(1,smp.col(i-1));
                P(smp.col(i)-1,smp.col(i)) = P(smp.col(i)-1,smp.col(i)) + 0.25*epsilon;
                P(smp.col(i)-1,smp.col(i)-1) = P(smp.col(i)-1,smp.col(i)-1) - 0.25*epsilon/3;
 %                    P(smp.col(i)-1,smp.col(i)-2)  =  P(smp.col(i)-1,smp.col(i)-2)  -  0.25*epsilon/3;
```

```
P(smp.col(i)-1,smp.col(i)-1+num_col) = P(smp.col(i)-1,smp.col(i)-1+num_col) - 0.25*epsilon/3;

                P(smp.col(i),smp.col(i)-1) = P(smp.col(i),smp.col(i)-1) - 0.25*epsilon;
                P(smp.col(i),smp.col(i)) = P(smp.col(i),smp.col(i)) + 0.25*epsilon/3;
                P(smp.col(i),smp.col(i)+1) = P(smp.col(i),smp.col(i)+1) + 0.25*epsilon/3;
                P(smp.col(i),smp.col(i)+num_col)        =        P(smp.col(i),smp.col(i)+num_col)        +
0.25*epsilon/3;

            elseif rand_series(i) >= 1/3 && rand_series(i)<2/3
                epsilon = data_std(1,smp.col(i)) - data_std(1,smp.col(i+1));
                P(smp.col(i)+1,smp.col(i)) = P(smp.col(i)+1,smp.col(i)) + 0.25*epsilon;
                P(smp.col(i)+1,smp.col(i)+1) = P(smp.col(i)+1,smp.col(i)+1) - 0.25*epsilon/3;
                P(smp.col(i)+1,smp.col(i)+2)    =    P(smp.col(i)+1,smp.col(i)+2)    -    0.25*epsilon/3;
P(smp.col(i)+1,smp.col(i)+1+num_col) = P(smp.col(i)+1,smp.col(i)+1+num_col) - 0.25*epsilon/3;

                P(smp.col(i),smp.col(i)+1) = P(smp.col(i),smp.col(i)+1) - 0.25*epsilon;
                P(smp.col(i),smp.col(i)) = P(smp.col(i),smp.col(i)) + 0.25*epsilon/3;
                P(smp.col(i),smp.col(i)-1) = P(smp.col(i),smp.col(i)-1) + 0.25*epsilon/3;
                P(smp.col(i),smp.col(i)+num_col)        =        P(smp.col(i),smp.col(i)+num_col)        +
0.25*epsilon/3;

            elseif rand_series(i) >= 2/3
                epsilon = data_std(1,smp.col(i)) - data_std(2,smp.col(i));
                P(smp.col(i)+num_col,smp.col(i))        =        P(smp.col(i)+num_col,smp.col(i))        +
0.25*epsilon;
                P(smp.col(i)+num_col,smp.col(i)+num_col*2)                                               =
P(smp.col(i)+num_col,smp.col(i)+num_col*2) - 0.25*epsilon/3;
                P(smp.col(i)+num_col,smp.col(i)+num_col-1)                                               =
P(smp.col(i)+num_col,smp.col(i)+num_col-1) - 0.25*epsilon/3;
                P(smp.col(i)+num_col,smp.col(i)+num_col+1)                                               =
P(smp.col(i)+num_col,smp.col(i)+num_col+1) - 0.25*epsilon/3;

                P(smp.col(i),smp.col(i)+num_col)        =        P(smp.col(i),smp.col(i)+num_col)        -
0.25*epsilon;
                P(smp.col(i),smp.col(i)) = P(smp.col(i),smp.col(i)) + 0.25*epsilon/3;
                P(smp.col(i),smp.col(i)-1) = P(smp.col(i),smp.col(i)-1) + 0.25*epsilon/3;
                P(smp.col(i),smp.col(i)+1) = P(smp.col(i),smp.col(i)+1) + 0.25*epsilon/3;
            end

        elseif smp.row(i) == num_row && smp.col(i) < num_col && smp.col(i) > 1                    if
rand_series(i) < 1/3
                epsilon = data_std(num_row,smp.col(i)) - data_std(num_row,smp.col(i-1));
                P((num_row-1)*num_col+smp.col(i)-1,(num_row-1)*num_col+smp.col(i))            =
P((num_row-1)*num_col+smp.col(i)-1,(num_row-1)*num_col+smp.col(i)) + 0.25*epsilon;
```

```
            P((num_row-1)*num_col+smp.col(i)-1,(num_row-1)*num_col+smp.col(i)-2) = P((num_row-1)*num_col+smp.col(i)-1,(num_row-1)*num_col+smp.col(i)-2) - 0.25*epsilon/3;
            P((num_row-1)*num_col+smp.col(i)-1,(num_row-1)*num_col+smp.col(i)-1) = P((num_row-1)*num_col+smp.col(i)-1,(num_row-1)*num_col+smp.col(i)-1) - 0.25*epsilon/3;
            P((num_row-1)*num_col+smp.col(i)-1,(num_row-2)*num_col+smp.col(i)-1) = P((num_row-1)*num_col+smp.col(i)-1,(num_row-2)*num_col+smp.col(i)-1) - 0.25*epsilon/3;

            P((num_row-1)*num_col+smp.col(i),(num_row-1)*num_col+smp.col(i)-1) = P((num_row-1)*num_col+smp.col(i),(num_row-1)*num_col+smp.col(i)-1) - 0.25*epsilon;
            P((num_row-1)*num_col+smp.col(i),(num_row-1)*num_col+smp.col(i)+1) = P((num_row-1)*num_col+smp.col(i),(num_row-1)*num_col+smp.col(i)+1) + 0.25*epsilon/3;
            P((num_row-1)*num_col+smp.col(i),(num_row-1)*num_col+smp.col(i)) = P((num_row-1)*num_col+smp.col(i),(num_row-1)*num_col+smp.col(i)) + 0.25*epsilon/3;
            P((num_row-1)*num_col+smp.col(i),(num_row-2)*num_col+smp.col(i)) = P((num_row-1)*num_col+smp.col(i),(num_row-2)*num_col+smp.col(i)) + 0.25*epsilon/3;

        elseif rand_series(i) >= 1/3 && rand_series(i)<2/3
            epsilon = data_std(num_row,smp.col(i)) - data_std(num_row,smp.col(i+1));
            P((num_row-1)*num_col+smp.col(i)+1,(num_row-1)*num_col+smp.col(i)) = P((num_row-1)*num_col+smp.col(i)+1,(num_row-1)*num_col+smp.col(i)) + 0.25*epsilon;
            P((num_row-1)*num_col+smp.col(i)+1,(num_row-1)*num_col+smp.col(i)+2) = P((num_row-1)*num_col+smp.col(i)+1,(num_row-1)*num_col+smp.col(i)+2) - 0.25*epsilon/3;
            P((num_row-1)*num_col+smp.col(i)+1,(num_row-1)*num_col+smp.col(i)+1) = P((num_row-1)*num_col+smp.col(i)+1,(num_row-1)*num_col+smp.col(i)+1) - 0.25*epsilon/3;
            P((num_row-1)*num_col+smp.col(i)+1,(num_row-2)*num_col+smp.col(i)+1) = P((num_row-1)*num_col+smp.col(i)+1,(num_row-2)*num_col+smp.col(i)+1) - 0.25*epsilon/3;

            P((num_row-1)*num_col+smp.col(i),(num_row-1)*num_col+smp.col(i)+1) = P((num_row-1)*num_col+smp.col(i),(num_row-1)*num_col+smp.col(i)+1) - 0.25*epsilon;
            P((num_row-1)*num_col+smp.col(i),(num_row-1)*num_col+smp.col(i)-1) = P((num_row-1)*num_col+smp.col(i),(num_row-1)*num_col+smp.col(i)-1) + 0.25*epsilon/3;
            P((num_row-1)*num_col+smp.col(i),(num_row-1)*num_col+smp.col(i)) = P((num_row-1)*num_col+smp.col(i),(num_row-1)*num_col+smp.col(i)) + 0.25*epsilon/3;
            P((num_row-1)*num_col+smp.col(i),(num_row-2)*num_col+smp.col(i)) = P((num_row-1)*num_col+smp.col(i),(num_row-2)*num_col+smp.col(i)) + 0.25*epsilon/3;

        elseif rand_series(i) >= 2/3
            epsilon = data_std(num_row,smp.col(i)) - data_std(num_row-1,smp.col(i));
            P((num_row-2)*num_col+smp.col(i),(num_row-1)*num_col+smp.col(i)) = P((num_row-2)*num_col+smp.col(i),(num_row-1)*num_col+smp.col(i)) + 0.25*epsilon;
            P((num_row-2)*num_col+smp.col(i),(num_row-2)*num_col+smp.col(i)-1) = P((num_row-2)*num_col+smp.col(i),(num_row-2)*num_col+smp.col(i)-1) - 0.25*epsilon/3;
            P((num_row-2)*num_col+smp.col(i),(num_row-2)*num_col+smp.col(i)+1) = P((num_row-2)*num_col+smp.col(i),(num_row-2)*num_col+smp.col(i)+1) - 0.25*epsilon/3;
```

```
                P((num_row-2)*num_col+smp.col(i),(num_row-3)*num_col+smp.col(i))                =
P((num_row-2)*num_col+smp.col(i),(num_row-3)*num_col+smp.col(i)) - 0.25*epsilon/3;

                P((num_row-1)*num_col+smp.col(i),(num_row-2)*num_col+smp.col(i))                =
P((num_row-1)*num_col+smp.col(i),(num_row-2)*num_col+smp.col(i)) - 0.25*epsilon;
                P((num_row-1)*num_col+smp.col(i),(num_row-1)*num_col+smp.col(i)-1)              =
P((num_row-1)*num_col+smp.col(i),(num_row-1)*num_col+smp.col(i)-1) + 0.25*epsilon/3;
                P((num_row-1)*num_col+smp.col(i),(num_row-1)*num_col+smp.col(i)+1)              =
P((num_row-1)*num_col+smp.col(i),(num_row-1)*num_col+smp.col(i)+1) + 0.25*epsilon/3;
                P((num_row-1)*num_col+smp.col(i),(num_row-1)*num_col+smp.col(i))                =
P((num_row-1)*num_col+smp.col(i),(num_row-1)*num_col+smp.col(i)) + 0.25*epsilon/3;

            end
        elseif smp.col(i) == 1 && smp.row(i) < num_row && smp.row(i) > 1
                epsilon = data_std(smp.row(i),smp.col(i)) - data_std(smp.row(i)-1,smp.col(i));
                P((smp.row(i)-2)*num_col+1,(smp.row(i)-1)*num_col+1)                            =
P((smp.row(i)-2)*num_col+1,(smp.row(i)-1)*num_col+1) + 0.25*epsilon;
                P((smp.row(i)-2)*num_col+1,(smp.row(i)-2)*num_col+1)                            =
P((smp.row(i)-2)*num_col+1,(smp.row(i)-2)*num_col+1) - 0.25*epsilon/3;
                P((smp.row(i)-2)*num_col+1,(smp.row(i)-2)*num_col+2)                            =
P((smp.row(i)-2)*num_col+1,(smp.row(i)-2)*num_col+2) - 0.25*epsilon/3;
                P((smp.row(i)-2)*num_col+1,(smp.row(i)-3)*num_col+1)                            =
P((smp.row(i)-2)*num_col+1,(smp.row(i)-3)*num_col+1) - 0.25*epsilon/3;

                P((smp.row(i)-1)*num_col+1,(smp.row(i)-2)*num_col+1)                            =
P((smp.row(i)-1)*num_col+1,(smp.row(i)-2)*num_col+1) - 0.25*epsilon;
                P((smp.row(i)-1)*num_col+1,(smp.row(i)-1)*num_col+1)                            =
P((smp.row(i)-1)*num_col+1,(smp.row(i)-1)*num_col+1) + 0.25*epsilon/3;
                P((smp.row(i)-1)*num_col+1,(smp.row(i)-1)*num_col+2)                            =
P((smp.row(i)-1)*num_col+1,(smp.row(i)-1)*num_col+2) + 0.25*epsilon/3;
                P((smp.row(i)-1)*num_col+1,(smp.row(i))*num_col+1)                              =
P((smp.row(i)-1)*num_col+1,(smp.row(i))*num_col+1) + 0.25*epsilon/3;

            elseif rand_series(i) >= 1/3 && rand_series(i)<2/3
                epsilon = data_std(smp.row(i),smp.col(i)) - data_std(smp.row(i),smp.col(i+1));
                P((smp.row(i))*num_col+1,(smp.row(i)-1)*num_col+1)                              =
P((smp.row(i))*num_col+1,(smp.row(i)-1)*num_col+1) + 0.25*epsilon;
                P((smp.row(i))*num_col+1,(smp.row(i))*num_col+1)                                =
P((smp.row(i))*num_col+1,(smp.row(i))*num_col+1) - 0.25*epsilon/3;
%                                   P((smp.row(i))*num_col+1,(smp.row(i+1))*num_col+1)          =
P((smp.row(i))*num_col+1,(smp.row(i+1))*num_col+1) - 0.25*epsilon/3;
                P((smp.row(i))*num_col+1,(smp.row(i))*num_col+2)                                =
P((smp.row(i))*num_col+1,(smp.row(i))*num_col+2) - 0.25*epsilon/3;
```

```
            P((smp.row(i)-1)*num_col+1,(smp.row(i)-2)*num_col+1)                    =
P((smp.row(i)-1)*num_col+1,(smp.row(i)-2)*num_col+1) - 0.25*epsilon;
            P((smp.row(i)-1)*num_col+1,(smp.row(i)-1)*num_col+1)                    =
P((smp.row(i)-1)*num_col+1,(smp.row(i)-1)*num_col+1) + 0.25*epsilon/3;
            P((smp.row(i)-1)*num_col+1,(smp.row(i)-1)*num_col+2)                    =
P((smp.row(i)-1)*num_col+1,(smp.row(i)-1)*num_col+2) + 0.25*epsilon/3;
            P((smp.row(i)-1)*num_col+1,(smp.row(i))*num_col+1)                      =
P((smp.row(i)-1)*num_col+1,(smp.row(i))*num_col+1) + 0.25*epsilon/3;

        elseif rand_series(i) >= 2/3
            epsilon = data_std(smp.row(i),smp.col(i)) - data_std(smp.row(i)+1,smp.col(i));
            P((smp.row(i)-1)*num_col+2,(smp.row(i)-1)*num_col+1)                    =
P((smp.row(i)-1)*num_col+2,(smp.row(i)-1)*num_col+1) + 0.25*epsilon;
            P((smp.row(i)-1)*num_col+2,(smp.row(i)-1)*num_col+3)                    =
P((smp.row(i)-1)*num_col+2,(smp.row(i)-1)*num_col+3) - 0.25*epsilon/3;
            P((smp.row(i)-1)*num_col+2,(smp.row(i)-2)*num_col+2)                    =
P((smp.row(i)-1)*num_col+2,(smp.row(i)-2)*num_col+2) - 0.25*epsilon/3;
            P((smp.row(i)-1)*num_col+2,(smp.row(i))*num_col+2)                      =
P((smp.row(i)-1)*num_col+2,(smp.row(i))*num_col+2) - 0.25*epsilon/3;

            P((smp.row(i)-1)*num_col+1,(smp.row(i)-1)*num_col+2)                    =
P((smp.row(i)-1)*num_col+1,(smp.row(i)-1)*num_col+2) - 0.25*epsilon;
            P((smp.row(i)-1)*num_col+1,(smp.row(i)-1)*num_col+1)                    =
P((smp.row(i)-1)*num_col+1,(smp.row(i)-1)*num_col+1) + 0.25*epsilon/3;
            P((smp.row(i)-1)*num_col+1,(smp.row(i))*num_col+1)                      =
P((smp.row(i)-1)*num_col+1,(smp.row(i))*num_col+1) + 0.25*epsilon/3;
            P((smp.row(i)-1)*num_col+1,(smp.row(i)-2)*num_col+1)                    =
P((smp.row(i)-1)*num_col+1,(smp.row(i)-2)*num_col+1) + 0.25*epsilon/3;

        end
    elseif smp.col(i) == num_col && smp.row(i) < num_row && smp.row(i) > 1
            epsilon = data_std(smp.row(i),smp.col(i)) - data_std(smp.row(i)-1,smp.col(i));
            P(smp.row(i)*num_col-1,smp.row(i)*num_col)                              =
P(smp.row(i)*num_col-1,smp.row(i)*num_col) + 0.25*epsilon;
            P(smp.row(i)*num_col-1,smp.row(i)*num_col-2)                            =
P(smp.row(i)*num_col-1,smp.row(i)*num_col-2) - 0.25*epsilon/3;
            P(smp.row(i)*num_col-1,(smp.row(i)-1)*num_col-1)                        =
P(smp.row(i)*num_col-1,(smp.row(i)-1)*num_col-1) - 0.25*epsilon/3;
            P(smp.row(i)*num_col-1,(smp.row(i)+1)*num_col-1)                        =
P(smp.row(i)*num_col-1,(smp.row(i)+1)*num_col-1) - 0.25*epsilon/3;

            P(smp.row(i)*num_col,smp.row(i)*num_col-1)                              =
P(smp.row(i)*num_col,smp.row(i)*num_col-1) - 0.25*epsilon;
            P(smp.row(i)*num_col,smp.row(i)*num_col)                                =
```

```
P(smp.row(i)*num_col,smp.row(i)*num_col) + 0.25*epsilon/3;
                P(smp.row(i)*num_col,(smp.row(i)-1)*num_col)                =
P(smp.row(i)*num_col,(smp.row(i)-1)*num_col) + 0.25*epsilon/3;
                P(smp.row(i)*num_col,(smp.row(i)+1)*num_col)                =
P(smp.row(i)*num_col,(smp.row(i)+1)*num_col) + 0.25*epsilon/3;


                epsilon = data_std(smp.row(i),smp.col(i)) - data_std(smp.row(i)-1,smp.col(i));
                P((smp.row(i)-1)*num_col,smp.row(i)*num_col)                =
P((smp.row(i)-1)*num_col,smp.row(i)*num_col) + 0.25*epsilon;
                P((smp.row(i)-1)*num_col,(smp.row(i)-1)*num_col)                =
P((smp.row(i)-1)*num_col,(smp.row(i)-1)*num_col) - 0.25*epsilon/3;
                P((smp.row(i)-1)*num_col,(smp.row(i)-1)*num_col-1)                =
P((smp.row(i)-1)*num_col,(smp.row(i)-1)*num_col-1) - 0.25*epsilon/3;
                P((smp.row(i)-1)*num_col,(smp.row(i)-2)*num_col)                =
P((smp.row(i)-1)*num_col,(smp.row(i)-2)*num_col) - 0.25*epsilon/3;
                        P(smp.row(i)*num_col,(smp.row(i)-1)*num_col)                =
P(smp.row(i)*num_col,(smp.row(i)-1)*num_col) - 0.25*epsilon;
                P(smp.row(i)*num_col,smp.row(i)*num_col)                =
P(smp.row(i)*num_col,smp.row(i)*num_col) + 0.25*epsilon/3;
                P(smp.row(i)*num_col,(smp.row(i)+1)*num_col)                =
P(smp.row(i)*num_col,(smp.row(i)+1)*num_col) + 0.25*epsilon/3;
                P(smp.row(i)*num_col,smp.row(i)*num_col)                =
P(smp.row(i)*num_col,smp.row(i)*num_col) + 0.25*epsilon/3;

            elseif rand_series(i) >= 2/3
                epsilon = data_std(smp.row(i),smp.col(i)) - data_std(smp.row(i)+1,smp.col(i));
                P((smp.row(i)+1)*num_col,smp.row(i)*num_col)                =
P((smp.row(i)+1)*num_col,smp.row(i)*num_col) + 0.25*epsilon;
                P((smp.row(i)+1)*num_col,(smp.row(i)+1)*num_col)                =
P((smp.row(i)+1)*num_col,(smp.row(i)+1)*num_col) - 0.25*epsilon/3;
                P((smp.row(i)+1)*num_col,(smp.row(i)+1)*num_col-1)                =
P((smp.row(i)+1)*num_col,(smp.row(i)+1)*num_col-1) - 0.25*epsilon/3;
                P((smp.row(i)+1)*num_col,(smp.row(i)+2)*num_col)                =
P((smp.row(i)+1)*num_col,(smp.row(i)+2)*num_col) - 0.25*epsilon/3;
                        P(smp.row(i)*num_col,(smp.row(i)+1)*num_col)                =
P(smp.row(i)*num_col,(smp.row(i)+1)*num_col) - 0.25*epsilon;
                P(smp.row(i)*num_col,smp.row(i)*num_col)                =
P(smp.row(i)*num_col,smp.row(i)*num_col) + 0.25*epsilon/3;
                P(smp.row(i)*num_col,smp.row(i)*num_col-1)                =
P(smp.row(i)*num_col,smp.row(i)*num_col-1) + 0.25*epsilon/3;
                P(smp.row(i)*num_col,(smp.row(i)+1)*num_col)                =
P(smp.row(i)*num_col,(smp.row(i)+1)*num_col) + 0.25*epsilon/3;
```

```
            end
        elseif smp.col(i) ~= 1 && smp.col(i) ~= num_col && smp.row(i) ~= 1 && smp.row(i) ~= 
num_row
      %elseif smp.col(i) ~= 1 && smp.col(i) < 9    && smp.row(i) ~= 1 && smp.row(i)<9
            if rand_series(i) < 0.25
                epsilon = data_std(smp.row(i),smp.col(i)) - data_std(smp.row(i),smp.col(i-1));
                P((smp.row(i)-2)*num_col+smp.col(i),(smp.row(i)-1)*num_col+smp.col(i)) = ...
                    P((smp.row(i)-2)*num_col+smp.col(i),(smp.row(i)-1)*num_col+smp.col(i))   + 
0.25*epsilon;
                P((smp.row(i)-2)*num_col+smp.col(i),(smp.row(i)-2)*num_col+smp.col(i)+1) = ...
                    P((smp.row(i)-2)*num_col+smp.col(i),(smp.row(i)-2)*num_col+smp.col(i)+1) 
- 0.25*epsilon/3;
                P((smp.row(i)-2)*num_col+smp.col(i),(smp.row(i)-2)*num_col+smp.col(i)-1) = ...
                    P((smp.row(i)-2)*num_col+smp.col(i),(smp.row(i)-2)*num_col+smp.col(i)-1) 
- 0.25*epsilon/3;
                P((smp.row(i)-2)*num_col+smp.col(i),(smp.row(i)-3)*num_col+smp.col(i)) = ...
                    P((smp.row(i)-2)*num_col+smp.col(i),(smp.row(i)-3)*num_col+smp.col(i))   - 
0.25*epsilon/3;

                P((smp.row(i)-1)*num_col+smp.col(i),(smp.row(i)-2)*num_col+smp.col(i)) = ...
                    P((smp.row(i)-1)*num_col+smp.col(i),(smp.row(i)-2)*num_col+smp.col(i))   - 
0.25*epsilon;
                P((smp.row(i)-1)*num_col+smp.col(i),(smp.row(i))*num_col+smp.col(i)) = ...
                    P((smp.row(i)-1)*num_col+smp.col(i),(smp.row(i))*num_col+smp.col(i))     + 
0.25*epsilon/3;
                P((smp.row(i)-1)*num_col+smp.col(i),(smp.row(i)-1)*num_col+smp.col(i)+1) = ...
                    P((smp.row(i)-1)*num_col+smp.col(i),(smp.row(i)-1)*num_col+smp.col(i)+1) 
+ 0.25*epsilon/3;
                P((smp.row(i)-1)*num_col+smp.col(i),(smp.row(i)-1)*num_col+smp.col(i)-1) = ...
                    P((smp.row(i)-1)*num_col+smp.col(i),(smp.row(i)-1)*num_col+smp.col(i)-1) 
+ 0.25*epsilon/3;

            elseif rand_series(i) >= 0.25 && rand_series(i) < 0.5
                epsilon = data_std(smp.row(i),smp.col(i)) - data_std(smp.row(i),smp.col(i+1));
                P((smp.row(i))*num_col+smp.col(i),(smp.row(i)-1)*num_col+smp.col(i)) = ...
                    P((smp.row(i))*num_col+smp.col(i),(smp.row(i)-1)*num_col+smp.col(i))     + 
0.25*epsilon;
                P((smp.row(i))*num_col+smp.col(i),(smp.row(i))*num_col+smp.col(i)+1) = ...
                    P((smp.row(i))*num_col+smp.col(i),(smp.row(i))*num_col+smp.col(i)+1)     - 
0.25*epsilon/3;
                P((smp.row(i))*num_col+smp.col(i),(smp.row(i))*num_col+smp.col(i)-1) = ...
                    P((smp.row(i))*num_col+smp.col(i),(smp.row(i))*num_col+smp.col(i)-1)     - 
0.25*epsilon/3;
                P((smp.row(i))*num_col+smp.col(i),(smp.row(i)+1)*num_col+smp.col(i)) = ...
```

```
                        P((smp.row(i))*num_col+smp.col(i),(smp.row(i)+1)*num_col+smp.col(i))      -
0.25*epsilon/3;

                    P((smp.row(i)-1)*num_col+smp.col(i),(smp.row(i))*num_col+smp.col(i)) = ...
                        P((smp.row(i)-1)*num_col+smp.col(i),(smp.row(i))*num_col+smp.col(i))       -
0.25*epsilon;
                    P((smp.row(i)-1)*num_col+smp.col(i),(smp.row(i)-1)*num_col+smp.col(i)-1) = ...
                        P((smp.row(i)-1)*num_col+smp.col(i),(smp.row(i)-1)*num_col+smp.col(i)-1)
+ 0.25*epsilon/3;
                    P((smp.row(i)-1)*num_col+smp.col(i),(smp.row(i)-1)*num_col+smp.col(i)+1) = ...
                        P((smp.row(i)-1)*num_col+smp.col(i),(smp.row(i)-1)*num_col+smp.col(i)+1)
+ 0.25*epsilon/3;
                    P((smp.row(i)-1)*num_col+smp.col(i),(smp.row(i)-2)*num_col+smp.col(i)) = ...
                        P((smp.row(i)-1)*num_col+smp.col(i),(smp.row(i)-2)*num_col+smp.col(i))   +
0.25*epsilon/3;

                elseif rand_series(i) >= 0.5 && rand_series(i) < 0.75
                    epsilon = data_std(smp.row(i),smp.col(i)) - data_std(smp.row(i-1),smp.col(i));
                    P((smp.row(i)-1)*num_col+smp.col(i)-1,(smp.row(i)-1)*num_col+smp.col(i)) = ...
                        P((smp.row(i)-1)*num_col+smp.col(i)-1,(smp.row(i)-1)*num_col+smp.col(i))
+ 0.25*epsilon;
                    P((smp.row(i)-1)*num_col+smp.col(i)-1,(smp.row(i)-1)*num_col+smp.col(i)+2)
= ...

P((smp.row(i)-1)*num_col+smp.col(i)-1,(smp.row(i)-1)*num_col+smp.col(i)+2) - 0.25*epsilon/3;
                    P((smp.row(i)-1)*num_col+smp.col(i)-1,(smp.row(i))*num_col+smp.col(i)+1) = ...
                        P((smp.row(i)-1)*num_col+smp.col(i)-1,(smp.row(i))*num_col+smp.col(i)+1)
- 0.25*epsilon/3;
                    P((smp.row(i)-1)*num_col+smp.col(i)-1,(smp.row(i)-2)*num_col+smp.col(i)+1)
= ...

P((smp.row(i)-1)*num_col+smp.col(i)-1,(smp.row(i)-2)*num_col+smp.col(i)+1) - 0.25*epsilon/3;

                    P((smp.row(i)-1)*num_col+smp.col(i),(smp.row(i)-1)*num_col+smp.col(i)-1) = ...
                        P((smp.row(i)-1)*num_col+smp.col(i),(smp.row(i)-1)*num_col+smp.col(i)-1)
- 0.25*epsilon;
                    P((smp.row(i)-1)*num_col+smp.col(i),(smp.row(i)-1)*num_col+smp.col(i)+1) = ...
                        P((smp.row(i)-1)*num_col+smp.col(i),(smp.row(i)-1)*num_col+smp.col(i)+1)
+ 0.25*epsilon/3;
                    P((smp.row(i)-1)*num_col+smp.col(i),(smp.row(i))*num_col+smp.col(i)) = ...
                        P((smp.row(i)-1)*num_col+smp.col(i),(smp.row(i))*num_col+smp.col(i))       +
0.25*epsilon/3;
                    P((smp.row(i)-1)*num_col+smp.col(i),(smp.row(i)-2)*num_col+smp.col(i)) = ...
                        P((smp.row(i)-1)*num_col+smp.col(i),(smp.row(i)-2)*num_col+smp.col(i))   +
```

```
0.25*epsilon/3;

                elseif rand_series(i) >= 0.75
                    epsilon = data_std(smp.row(i),smp.col(i)) - data_std(smp.row(i+1),smp.col(i));
                    P((smp.row(i)-1)*num_col+smp.col(i)+1,(smp.row(i)-1)*num_col+smp.col(i)) = ...
                        P((smp.row(i)-1)*num_col+smp.col(i)+1,(smp.row(i)-1)*num_col+smp.col(i))
+ 0.25*epsilon;
                    P((smp.row(i)-1)*num_col+smp.col(i)+1,(smp.row(i)-1)*num_col+smp.col(i)+2)
= ...

P((smp.row(i)-1)*num_col+smp.col(i)+1,(smp.row(i)-1)*num_col+smp.col(i)+2) - 0.25*epsilon/3;
                    P((smp.row(i)-1)*num_col+smp.col(i)+1,(smp.row(i))*num_col+smp.col(i)+1) = ...
                        P((smp.row(i)-1)*num_col+smp.col(i)+1,(smp.row(i))*num_col+smp.col(i)+1)
- 0.25*epsilon/3;
                    P((smp.row(i)-1)*num_col+smp.col(i)+1,(smp.row(i)-2)*num_col+smp.col(i)+1)
= ...

P((smp.row(i)-1)*num_col+smp.col(i)+1,(smp.row(i)-2)*num_col+smp.col(i)+1) - 0.25*epsilon/3;

                    P((smp.row(i)-1)*num_col+smp.col(i),(smp.row(i)-1)*num_col+smp.col(i)+1) = ...
                        P((smp.row(i)-1)*num_col+smp.col(i),(smp.row(i)-1)*num_col+smp.col(i)+1)
- 0.25*epsilon;
                    P((smp.row(i)-1)*num_col+smp.col(i),(smp.row(i)-1)*num_col+smp.col(i)-1) = ...
                        P((smp.row(i)-1)*num_col+smp.col(i),(smp.row(i)-1)*num_col+smp.col(i)-1)
+ 0.25*epsilon/3;
                    P((smp.row(i)-1)*num_col+smp.col(i),(smp.row(i))*num_col+smp.col(i)) = ...
                        P((smp.row(i)-1)*num_col+smp.col(i),(smp.row(i))*num_col+smp.col(i))      +
0.25*epsilon/3;
                    P((smp.row(i)-1)*num_col+smp.col(i),(smp.row(i)-2)*num_col+smp.col(i)) = ...
                        P((smp.row(i)-1)*num_col+smp.col(i),(smp.row(i)-2)*num_col+smp.col(i))   +
0.25*epsilon/3;
                end
        end
end

pi = fun_staEqu(P);
pi_surf = reshape(pi,num_col,num_row)';
max_pi_surf=max(pi);
[rows, cols]=find(pi==max(pi));
hang=ceil(cols/9);

if mod(cols,9)~=0
        lie=rem(cols,9);
else lie=9;
```

```
end

figure
surf(x,y,pi_surf);
con={'0','0.1','1','5','10','50','100','1000','5000'};
con1={'5000','1000','100','50','10','5','1','0.1','0'};
set(gca, 'XtickLabel',con);
set(gca, 'YtickLabel',con);
xlabel('DOX-concentration(nM)')
ylabel('PTX-concentration(nM)')
zlabel('pi')
max_pi_surf
[rows,cols]
[hang,lie]
num_smpPer
data_std(hang,lie)

function pi = fun_staEqu(P)
I = eye(size(P,1));
A0 = (I-P)';
A = [A0;ones(1,size(P,1))];
B = [zeros(size(P,1),1);1];
x = A\B;
pi = x';
end
```
