# Supplementary figures and images for: Concentration optimization of combinatorial drugs using Markov chain-based models
Source: BMC Bioinformatics. 2021 Sep 21;22:451. doi: 10.1186/s12859-021-04364-5 (PMC8456646; doi:10.1186/s12859-021-04364-5)

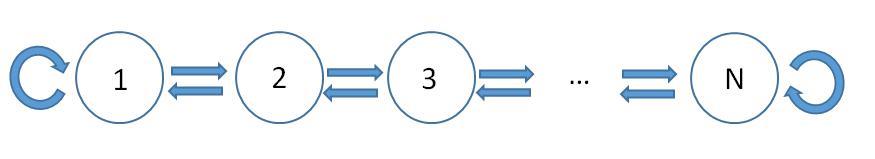

Supplement: Supplementary file 2 — Additional file 2. Figure S1. State-transition diagram of an Markov chain. [file 12859_2021_4364_MOESM2_ESM.jpg]

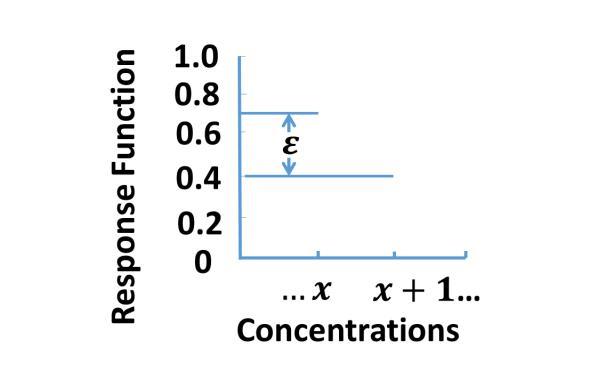

Supplement: Supplementary file 3 — Additional file 3. Figure S2. Drug response function between two adjacent concentrations. [file 12859_2021_4364_MOESM3_ESM.jpg]

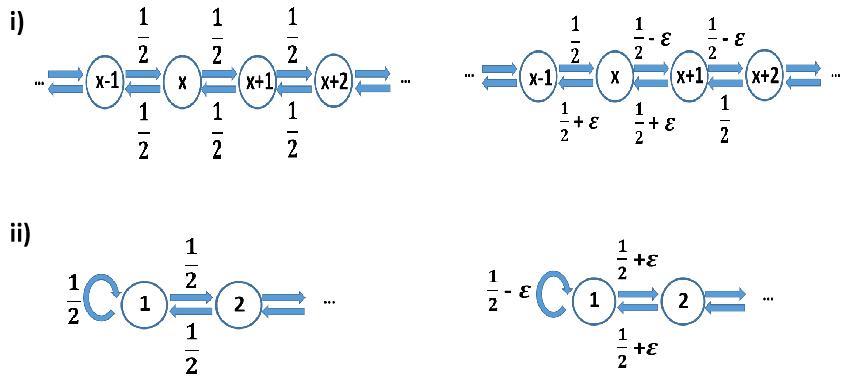

Supplement: Supplementary file 4 — Additional file 4. Figure S3. Initializing the Markov chain and updating the corresponding transition probability according to two adjacent experimental points. [file 12859_2021_4364_MOESM4_ESM.jpg]

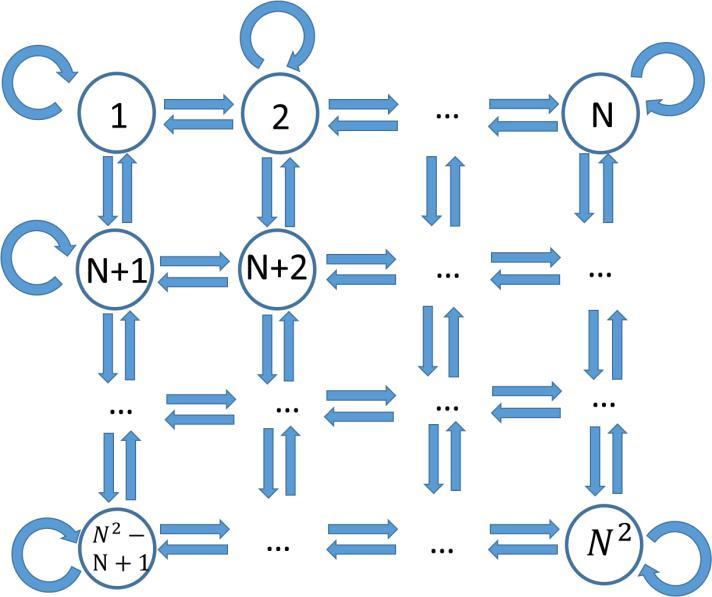

Supplement: Supplementary file 5 — Additional file 5. Figure S4. Two-drug case: a two-dimensional network structure with N2 states. [file 12859_2021_4364_MOESM5_ESM.jpg]

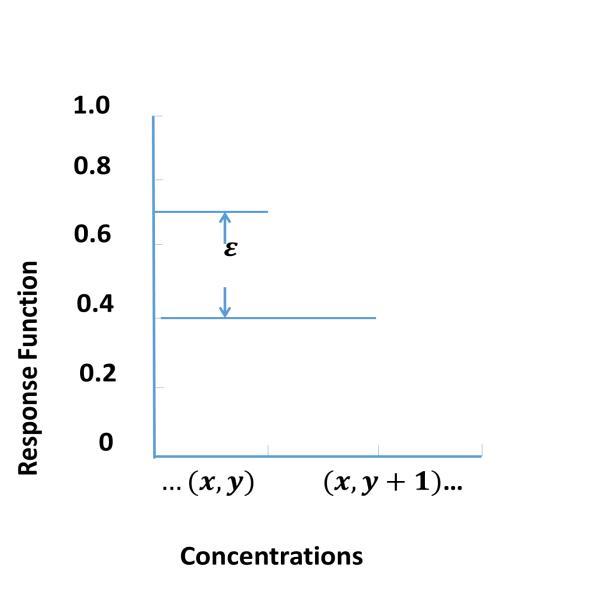

Supplement: Supplementary file 6 — Additional file 6. Figure S5. Drug response function at concentration levels (x,y) and (x,y+1). [file 12859_2021_4364_MOESM6_ESM.jpg]

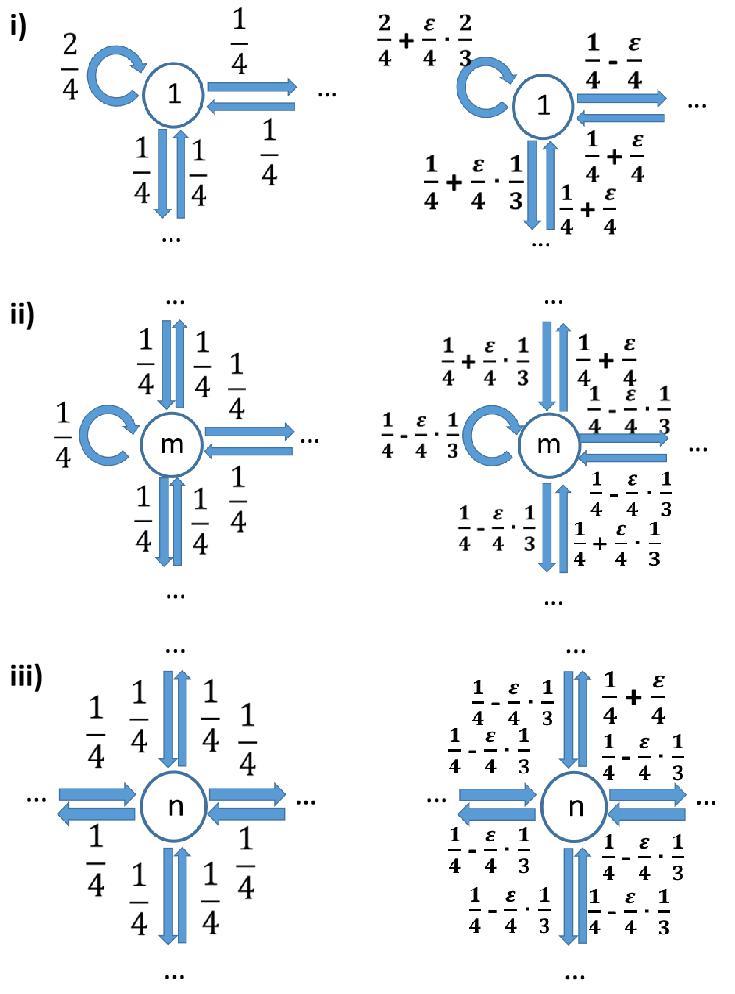

Supplement: Supplementary file 7 — Additional file 7. Figure S6. Initializing the Markov chain and updating the corresponding Markov-chain-based transition probability based on the two neighboring states. [file 12859_2021_4364_MOESM7_ESM.jpg]
